# Prediction of long-term humoral response induced by the two-dose heterologous Ad26.ZEBOV, MVA-BN-Filo vaccine against Ebola

**DOI:** 10.1038/s41541-023-00767-y

**Published:** 2023-11-08

**Authors:** Marie Alexandre, Mélanie Prague, Chelsea McLean, Viki Bockstal, Macaya Douoguih, Rodolphe Thiébaut, for the EBOVAC 1 and EBOVAC 2 Consortia, Thierry Van Effelterre, Laura Solforosi, Viki Bockstal, Anna Dari

**Affiliations:** 1 Department of Public Health, Bordeaux University, Inserm UMR 1219 Bordeaux Population Health Research Center, Inria SISTM, Bordeaux, France; 2 Vaccine Research Institute, Créteil, France; 3 Janssen Vaccines and Prevention, Leiden, the Netherlands; 4 Janssen Pharmaceutica N.V., Beerse, Belgium; 5 Janssen Research and Development, Beerse, Belgium; 6 Present Address: ExeVir, Ghent, Belgium; 7 Present Address: Janssen Pharmaceutica N.V., Global Commercial Strategy Organization, Beerse, Belgium

**Keywords:** Vaccines, Viral infection, Humoral immunity

## Abstract

The persistence of the long-term immune response induced by the heterologous Ad26.ZEBOV, MVA-BN-Filo two-dose vaccination regimen against Ebola has been investigated in several clinical trials. Longitudinal data on IgG-binding antibody concentrations were analyzed from 487 participants enrolled in six Phase I and Phase II clinical trials conducted by the EBOVAC1 and EBOVAC2 consortia. A model based on ordinary differential equations describing the dynamics of antibodies and short- and long-lived antibody-secreting cells (ASCs) was used to model the humoral response from 7 days after the second vaccination to a follow-up period of 2 years. Using a population-based approach, we first assessed the robustness of the model, which was originally estimated based on Phase I data, against all data. Then we assessed the longevity of the humoral response and identified factors that influence these dynamics. We estimated a half-life of the long-lived ASC of at least 15 years and found an influence of geographic region, sex, and age on the humoral response dynamics, with longer antibody persistence in Europeans and women and higher production of antibodies in younger participants.

## Introduction

The 2014–2016 Ebola virus disease (EBOV) outbreak in West Africa and the current SARS-CoV-2 pandemic have led to accelerated development of vaccines to control the spread of infection and reduce the severity of disease in infected individuals. As a result, effective vaccines were developed and became available quickly after the start of these two epidemics. In the case of Ebola, the recombinant replication-competent vesicular stomatitis viral vectored vaccine (Ervebo) was approved by the FDA in December 2019^1^ and used during epidemics in a ring vaccination strategy. The two-dose heterologous strategy, combining immunizations with Ad26.ZEBOV (Zabdeno) and MVA-BN-Filo (Mvabea), were approved by the European Commission in July 2020^2^ under exceptional circumstances for use in children and adults. An important question for those who have already been vaccinated, and for using the vaccines as a preventive strategy to control the occurrence of outbreaks, is the duration of protection conferred by vaccination.

In the context of rapid vaccine development, long-term follow-up in large populations of vaccinated persons, as with older vaccines, is not possible^3,4^. When data are sparse, mathematical modeling is helpful because it can provide estimates of the duration of response by using additional information from biological knowledge about the vaccine mechanism and biological parameters. It is also helpful in quantifying the effect of factors that influence the response to the vaccine. This type of work is performed by modeling the dynamics of one or several markers that could be considered good correlates of protection^5^. Vaccine efficacy and mechanisms of action, or optimal immunogenic vaccine doses, have been evaluated for various infectious diseases, such as influenza^6–8^, yellow fever^9,10^, Zika^11^, tuberculosis^12^, and more recently SARS-CoV-2^13^. In the case of the Ad26.ZEBOV and MVA-BN-Filo vaccine strategy, the concentration of binding antibodies is considered a good correlate of protection based on work performed in non-human primates^14^. It was agreed with the FDA to be suitable for use in a Biological Licensing Application under the Animal Rule^15^ and it was the basis for marketing authorization in the EU.

In a previous publication^16^, we used a mathematical model for antibody-secreting cell (ASC) dynamics that distinguishes between short-lived and long-lived cells (SL and LL, respectively), and we estimated the model parameters using the data from the available first Phase I studies. We found that antibody production is maintained by the population of long-lived cells with an estimated half-life of at least 5 years. New data from three Phase II studies^17–19^ conducted in two international consortia (EBOVAC1 and EBOVAC2) provided an opportunity to validate the model and better characterize factors associated with the variation of the antibody response.

## Results

### Descriptive analysis of the data

The baseline and demographic characteristics of the 487 participants included in the study are shown in Table 1. In all results hereafter, Benjamini and Hochberg correction^20^ for multiple testing has been used (see the section “Methods” for more details). Comparable baseline characteristics in terms of age, body mass index (BMI), and weight are observed in European participants across the Phase I and II clinical studies (all *p*-values > 0.80). Similarly, no differences are observed in Africa across trials and sites in terms of weight, however, BMI appears significantly higher in East African participants (+6%, *p*-value = 0.007) than in West African ones. European participants were significantly older than Africans (41 vs. 29 years, *p*-value < 0.001) and consequently, participants in EBL2002 tended to be older (34 vs. 27 years, *p*-value < 0.001). BMI and weight (*p*-values < 0.001 in both cases) were significantly higher in European participants (+13% and +18%, respectively) than in African participants.

**Table 1:** Demographic and baseline characteristics of participants.

	Phase I trials			Phase II trials				Total
	Europe	East Africa		Europe	East Africa	West Africa	West Africa	
	UK	Kenya	Tanz./Ug.	UK/France	Ken./Tanz.	BFA/IVC	Sierra Leone	
	EBL1001	EBL1003	EBL1004	EBL2001	EBL2002		EBL3001	
Part., no.	14	15	15	71	79	58	235	487
Sex								
Men	4 (29%)	11 (73%)	10 (67%)	32 (45%)	45 (57%)	44 (75%)	203 (86%)	349 (72%)
Women	10 (71%)	4 (27%)	5 (33%)	39 (55%)	34 (43%)	14 (24%)	32 (14%)	138 (28%)
Age (yrs)	37.6 (9.3)	23.7 (2.8)	26.5 (6.8)	41.2 (14.7)	34.1 (13.5)	34.1 (10.8)	27.2 (10.0)	31.3 (12.4)
BMI (kg/m^2^)	26.1 (3.3)	22.5 (4.1)	22.9 (4.2)	25.4 (4.5)	23.8 (4.0)	23.0 (3.4)	21.9 (3.3)	23.0 (3.9)
Weight (kg)	73.7 (13.7)	63.3 (12.7)	63.5 (11.7)	74.7 (14.7)	63.9 (10.2)	67.2 (9.9)	62.4 (9.4)	65.4 (11.7)

Data are *n* (%) or mean (SD). Only healthy adults receiving Ad26.ZEBOV followed by MVA-BN-Filo 56 days later were selected within each of the 6 trials.

*Part.* participants, *no.* number, *yrs* years, *kg* kilograms, *m* meter, *UK* United Kingdom, *Tanz.* Tanzania, *Ug.* Uganda, *BFA* Burkina Faso, *IVC* Ivory Coast.

**Fig. 1:**
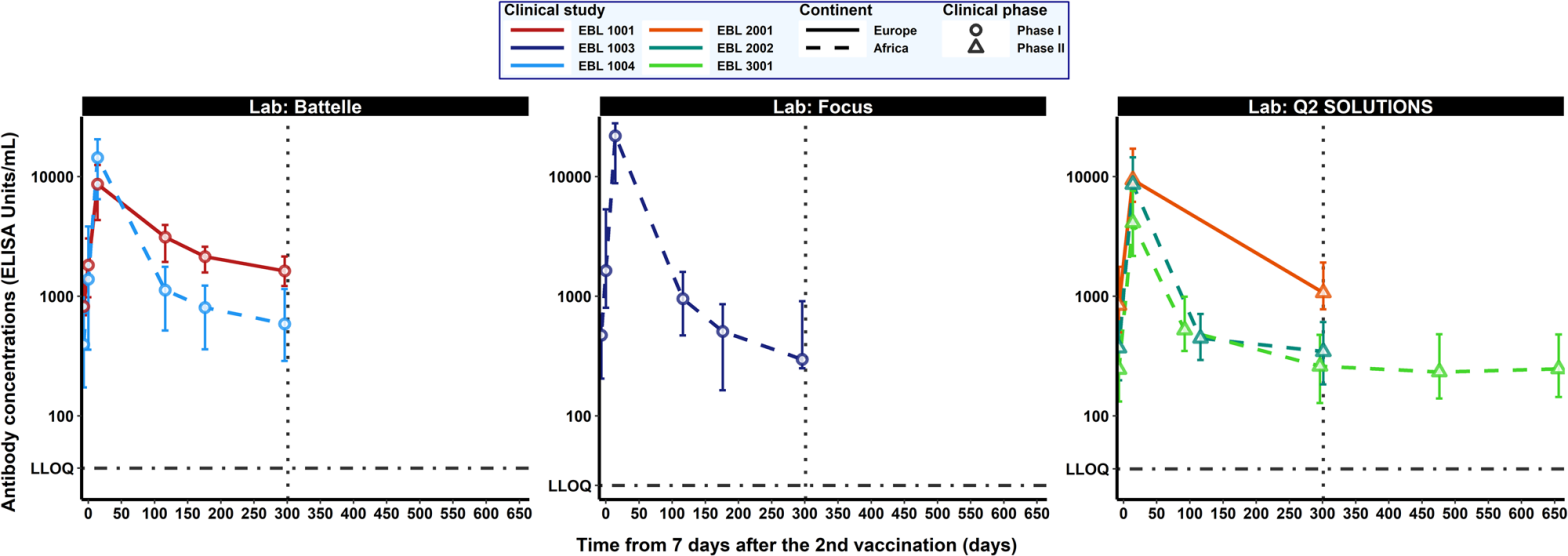
Dynamics of Ebola GP-specific binding antibody concentrations, in log_**10**_ scale (ELISA units/mL, EU/mL) for each clinical study from 7 days after the second vaccination. Each subplot represents the antibody dynamics measured in one of the three accredited laboratories: Battelle (left side), Focus (middle) and Q^2^ Solutions (right side). Each color corresponds to a clinical study (red: EBL1001, dark blue: EBL1003, light blue: EBL1004, orange: EBL2001, turquoise: EBL2002, light green: EBL3001). Solid and dashed lines represent medians in European and African participants, respectively. Circles correspond to Phase I studies and triangles to Phase II studies. Error bars correspond to 25th–75th confidence intervals. The vertical dotted line represents the first year after the first vaccination (309 days after the 2nd vaccination). The horizontal dot-dashed lines represent the LLOQ values considered for each laboratory (36.60, 26.22, and 36.11 EU/mL, at Battelle, Focus, and Q^2^ Solutions, respectively).

Figure 1 shows the dynamics of antibody concentrations (median and interquartile ranges) 7 days after the second vaccination for each study according to the assay used to quantify the binding antibodies. In addition, Table 2 summarizes antibody concentrations observed at predefined sampling time points. Only participants who had received both the first and second vaccinations were included in both the descriptive and the modeling analyses. Similar kinetics were observed in all studies, with the highest binding antibody concentrations observed at 21 days post-dose 2 (hereafter referred to as “peak"), followed by a biphasic decline up to 1 year after the first vaccination. Furthermore, the longer-term dynamics observed in EBL3001 suggest a durable immune response after the biphasic decline.

**Table 2:** Ebola glycoprotein-specific antibody concentrations (in log_10_ ELISA units/mL) in each trial from 7 days after the second vaccination to study completion.

	Phase I trials			Phase II trials		
	EBL1001	EBL1003	EBL1004	EBL2001	EBL2002	EBL3001
	(*n* = 14)^a^	(*n* = 15)^a^	(*n* = 15)^a^	(*n* = 71)^a^	(*n* = 137)^b^	(*n* = 235)^c^
*Day 64 (7 days after the 2nd vaccination, MVA-BN-Filo)*						
Number Part.	14	15	15			
Positive Part.^d^	14 (100%)	15 (100%)	15 (100%)			
Missing data	0 (0%)	0 (0%)	0 (0%)			
Mean [IQR]	3.19 [2.99; 3.48]	3.33 [2.90; 3.73]	3.09 [2.55; 3.58]			
*Day 78 (21 days after the 2nd vaccination, MVA-BN-Filo)*						
Number Part.	14	15	15	70	137	231
Positive Part.^d^	14 (100%)	15 (100%)	15 (100%)	70 (100%)	137 (100%)	231 (100%)
Missing data	0 (0%)	0 (0%)	0 (0%)	1 (1%)	0 (0%)	4 (2%)
Mean [IQR]	3.88 [3.64; 4.10]	4.21 [3.96; 4.45]	4.03 [3.80; 4.31]	4.00 [3.79; 4.43]	3.88 [3.62; 4.16]	3.60 [3.34; 3.88]
*Day 156 (155 days after the 1st vaccination, Ad26.ZEBOV)*						
Number Part.						42^e^
Positive Part.^d^						42 (100%)
Missing data						1 (2%)
Mean [IQR]						2.73 [2.54; 3.00]
*Day 180 (179 days after the 1st vaccination, Ad26.ZEBOV)*						
Number Part.	12	15	15		23^f^	
Positive Part.^d^	12 (100%)	15 (100%)	15 (100%)		23 (100%)	
Missing data	2 (14%)	0 (0%)	0 (0%)			
Mean [IQR]	3.47 [3.29; 3.60]	3.00 [2.67; 3.20]	2.97 [2.71; 3.25]		2.70 [2.47; 2.85]	
*Day 240 (239 days after the 1st vaccination, Ad26.ZEBOV)*						
Number Part.	13	15	15			
Positive Part.^d^	13 (100%)	15 (100%)	15 (100%)			
Missing data	1 (7%)	0 (0%)	0 (0%)			
Mean [IQR]	3.35 [3.20; 3.41]	2.66 [2.22; 2.93]	2.83 [2.56; 3.09]			
*Day 360/365 (1 year after the 1st vaccination, Ad26.ZEBOV)*						
Number Part.	12	15	15	51	134	207
Positive Part.^d^	12 (100%)	15 (100%)	15 (100%)	51 (100%)	134 (100%)	205 (99%)
Missing data	2 (14%)	0 (0%)	0 (0%)	20 (28%)	3 (2%)	28 (12%)
Mean [IQR]	3.24 [3.09; 3.33]	2.61 [2.40; 2.96]	2.74 [2.46; 3.06]	3.07 [2.89; 3.28]	2.54 [2.26; 2.78]	2.44 [2.11; 2.68]
*Day 540 (539 days after the 1st vaccination, Ad26.ZEBOV)*						
Number Part.						33^e^
Positive Part.^d^						33 (100%)
Missing data						10 (23%)
Mean [IQR]						2.43 [2.15; 2.68]
*Day 720 (2 years after the 1st vaccination, Ad26.ZEBOV)*						
Number Part.						190
Positive Part.^d^						184 (97%)
Missing data						45 (19 %)
Mean [IQR]						2.45 [2.19; 2.69]

IQR: Interquartile range = 75% confidence intervals, Part.: Participants.

^a^Participants receiving the 2nd vaccination in the protocol-defined window of 57 ± 1 day.

^b^Participants receiving the 2nd vaccination in the protocol-defined window of 57 ± 3 days.

^c^Participants receiving the 2nd vaccination in the protocol-defined window of 57 ± 1 week.

^d^Refers to the number of participants with antibody concentration above the lower limit of quantification (LLOQ), expressed as *n*/*N* (%) where *n* is the number of participants with a concentration above the LLOQ (i.e., 36.11 EU/mL for Battelle lab, 26.22 EU/mL for Focus lab and 36.11 EU/mL for Q^2^ Solutions lab) at that timepoint and *N* is the total number of participants with data at the first and the second vaccination and at that time point.

^e^Refers only to 43 participants enrolled in a substudy to receive a third dose (Ad26.ZEBOV) 2 years after the first vaccination.

^f^Refers to participants enrolled in EBL 2002 having an additional timepoint, initially scheduled for participants who do not receive a second vaccination because of a study pause.

### Mechanistic model of the humoral response

**Fig. 2:**
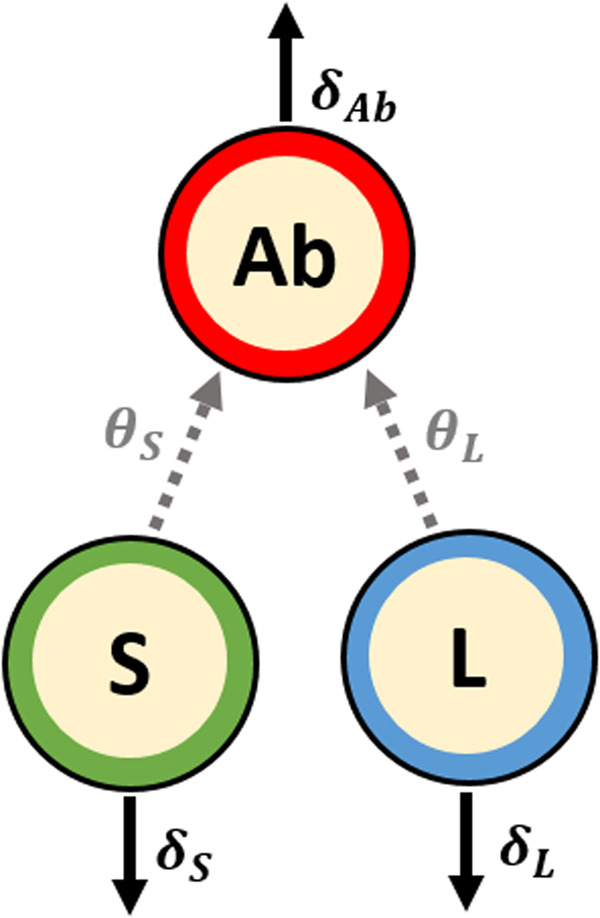
Schematic diagram of the model describing the humoral immune response from 7 days after the 2nd vaccination. S and L stand for short- and long-lived ASCs, respectively and *Ab* for antibodies. The parameters *δ*_S_, *δ*_L_, and *δ*_Ab_ are respectively the decay rates of SL ASCs, LL ASCs, and antibodies while *θ*_S_ and *θ*_L_ represent the production rates of antibodies by SL and LL ASCs.

To better identify the factors associated with the dynamic of the antibody response and to predict its duration, we used a model initially applied by Pasin et al.^16^ in Phase I trials evaluating the two-dose heterologous Ad26.ZEBOV, MVA-BN-Filo vaccine regimen. In this mechanistic model, antibodies are assumed to be produced by plasma cells (antibody-secreting cells, ASCs) divided into two distinct sub-populations characterized by their lifespan: short-lived (SL) and long-lived (LL). For various infectious diseases, a rapid expansion of antigen-specific ASCs in blood peaking on day 7 post-infection or vaccination, followed by a fast depletion is observed^21,22^. Therefore, strictly decreasing dynamics were considered from 7 days after the second vaccination for the two compartments of plasma cells assuming no additional exposure to the antigen. A schematic diagram of the mathematical model used to describe the humoral response from 7 days after the second vaccination is displayed in Fig. 2. This simple model relied on three biological processes. LL and SL ASCs decay with time at rate *δ*_L_ and *δ*_S_, respectively, and produce antibodies at rates *θ*_L_ and *θ*_S_, respectively. Finally, antibodies are assumed to decay over time at rate *δ*_Ab_. Since the baseline level of ASCs is unknown, the parameters *ϕ*_L_ = *θ*_L_*L*_0_ and *ϕ*_S_ = *θ*_S_*S*_0_ were defined, which represent the influx of LL and SL ASCs, respectively (see “Mathematical model of antibody kinetics” for more details).

### Quality of model prediction

Using parameter estimations obtained by Pasin et al.^16^ on humoral response observed in Phase I trials, we evaluated the robustness of the model and its predictive abilities.

First, we looked at the capacity of the model to capture the dynamic of the antibodies during the early phase following vaccination, based on the previously estimated parameters, in a new population of participants. Fixing the antibody, short- and long-lived ASCs half-lives at 24 days, 3.0 days and 6.0 years, respectively, as well as the SL ASC influx parameter at 2755 ELISA units/mL/day and the LL ASC influx parameter at 16.6 and 70.7 ELISA units/mL/day for African and European participants (see the “Methods” subsection “Evaluation of the model quality of prediction” for more details), only random effects (i.e. individual deviation from population mean) for the 487 participants were evaluated using empirical Bayes estimates (EBEs).

When restricted from 7 days post-second vaccination to the peak of individual dynamics, the model predictions fit well with observed antibody concentrations (see Table 3). The overall percent of observations falling within the 95% individual prediction intervals (also referred to as the percent of coverage) was evaluated at 100%. The root mean squared error (RMSE) was consistent with a relatively small average difference between observations and predictions estimated at 0.012 ${\log }_{10}$ ELISA units/ml (RMSE = 0.028 ${\log }_{10}$ ELISA units/ml in Phase I studies accounting for two observations). The model provided similar goodness of predictions when observations extended to the first year following vaccination were used to update EBEs (Supplementary Table 1). The percent of coverage was evaluated at 100% and the RMSE at 0.045 ${\log }_{10}$ ELISA units/ml (RMSE = 0.066 and 0.038 ${\log }_{10}$ ELISA units/ml in Phase I and Phase II studies, respectively). These results confirmed the ability of the model, estimated using only data from Phase I trials, to capture the antibody response in all additional participants included in Phase II trials.

**Table 3:** Evaluation of the robustness and the quality of prediction of the model developed by Pasin et al.^16^.

	All trials	Phase I trials			Phase II trials		
		EBL1001	EBL1003	EBL1004	EBL2001	EBL2002	EBL3001
Time of peak^a^							
Mean [95% CI]	15.0 [5.0; 21.0]	13.4 [11.6; 14.6]	14.4 [13.0; 18.2]	13.4 [12.0; 14.0]	14.3 [13.0; 16.3]	14.0 [12.4; 15.0]	16.0 [3.9; 22.2]
Number of timepoints^b^							
Mean [Min–Max]	1.09 [1.0; 2.0]	2.0 [2.0; 2.0]	2.0 [2.0; 2.0]	2.0 [2.0; 2.0]	1.0 [1.0; 1.0]	1.0 [1.0; 1.0]	1.0 [1.0; 1.0]
*Predictions from 7 days post-2nd vaccination to the peak*							
RMSE^c^	0.012	0.034	0.023	0.026	0.005	0.006	0.006
Coverage (%)	100	100	100	100	100	100	100
Bias^c^	0.002	0.018	-0.003	0.006	0.001	0.001	0.000
95% PI width	0.410	0.547	0.502	0.510	0.389	0.389	0.387
*Short-term forecast from the peak to 1 year*							
RMSE^c^	0.471	0.218	0.539	0.361	0.442	0.460	0.515
Coverage (%)	98.1	100	93.3	100	100	98.7	97.6
Bias^c^	0.251	0.014	0.239	0.077	0.293	0.224	0.328
95% PI width	2.297	2.097	2.124	2.184	2.502	2.358	2.298
*Long-term forecast beyond 1 year*							
RMSE^c^	0.518						0.518
Coverage (%)	97.8						97.8
Bias^c^	0.253						0.253
95% PI width	2.479						2.479

The model was estimated on Phase I data and individual parameters were assessed, for each participant of Phase I and Phase II trials, using observation from 7 days post-second vaccination (day 64) to the peak of individual dynamics.

*CI* confidence interval, *PI* prediction interval, *RMSE* root mean squared error.

^a^Time delay in days (Mean [95% CI]) from 7 days post-vaccination (day 64) to the peak (first local maximum).

^b^Number of observations from 7 days post-vaccination to the peak.

^c^Criteria calculated on the median of individual predictions.

**Fig. 3:**
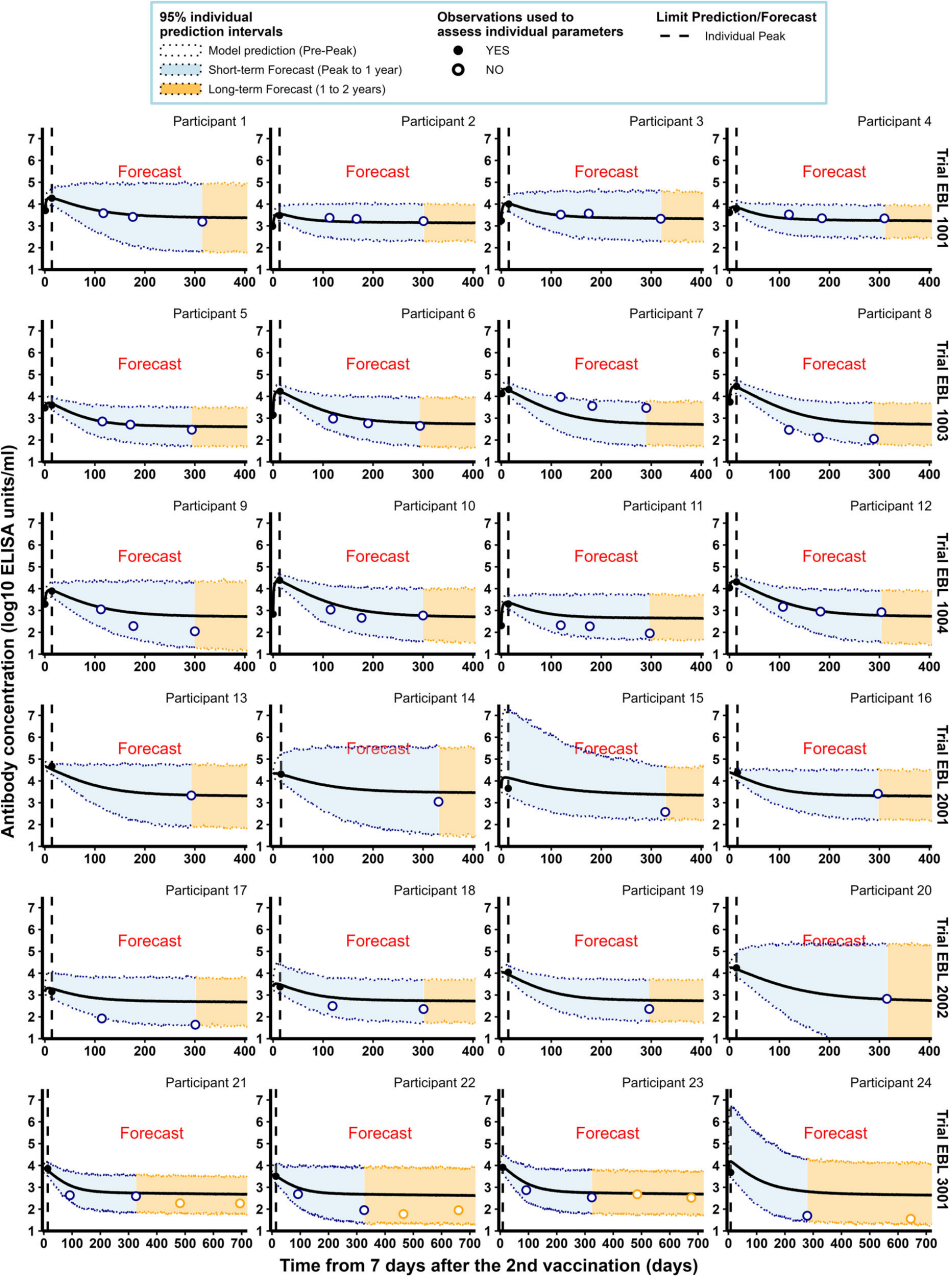
Individual antibody concentrations predicted by the model, estimated on Phase I data, for a random sample of participants from the six clinical studies. Each subplot represents the individual antibody dynamics (in log_10_ ELISA units/mL) from 7 days after the 2nd vaccination. For each participant, the vertical dashed line represents the time limit (individual peak of dynamics) between the predictions (on the left) and the forecasts (short-term in blue and long-term in orange). Plain dots correspond to observations used to evaluate individual parameters while circles are observations not used in parameter estimation. Shaded areas correspond to 95% individual prediction intervals (accounting for the uncertainty on the individual parameter estimation and the measurement error) and the solid lines correspond to the prediction of the model.

Then, we looked at the ability of the model to predict antibody concentrations beyond the peak of the dynamics. Individual parameters assessed for the early part of the dynamics of humoral responses were then used to predict both short-term antibody responses between the peak and 1 year after the first vaccination, and long-term antibody responses between 1 year and 2 years. As described in Table 2, while participants from all trials were included in the analysis of short-term predictions, only participants from the EBL3001 clinical trial contributed to the analysis of long-term predictions because they were the only ones with a follow-up beyond 12 months. As shown in Fig. 3 and Table 3, the model demonstrated a high quality of short- and long-term predictions with a total of 98% of the observed antibody concentrations falling within the 95% individual prediction intervals. Nevertheless, the high width of 95% prediction intervals (2.297 and 2.479 ${\log }_{10}$ ELISA units/mL for short- and long-term forecast, respectively) highlights a large uncertainty in individual model parameters and explains the high percentage of coverage. Plots of individual predictions for more participants having at least two observations from 7 days post-second vaccination are given in Supplementary Figs. 1–3. Similar work was done to evaluate the ability of the model to predict long-term antibody concentrations beyond 12 months when data from 7 days post-second vaccination to 1 year were used to estimate individual parameters (see Supplementary Figs. 4–6). These results highlight the benefit of using an additional short-term observation to improve long-term predictions (beyond 1 year after the second vaccination). The uncertainty of predictions was much lower (65% reduction in the size of prediction intervals) leading to a fair but smaller coverage (90% vs. 98%) and a significant improvement in the quality of the predictions (RMSE: 0.298 instead of 0.518; bias: −0.017 instead of 0.253).

### Additional insight on longevity of the humoral immune response

**Fig. 4:**
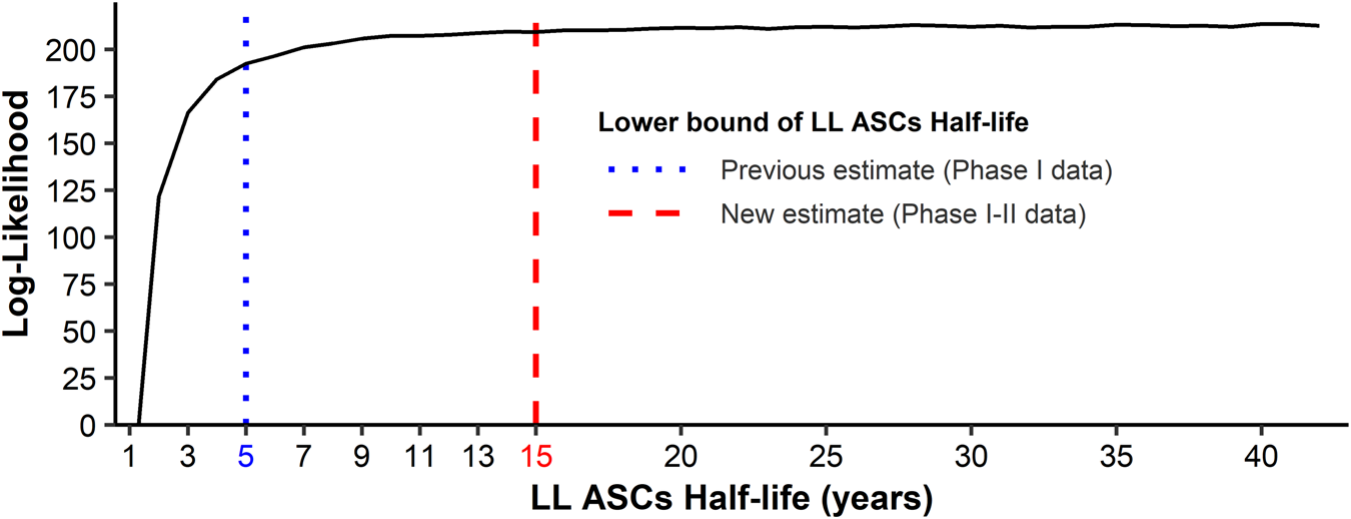
Profile likelihood on parameter ***δ***_**L**_. The *y*-axis corresponds to the non-penalized log-likelihood computed by importance sampling for several values of LL ASCs half-life which needs to be maximized. The blue dotted vertical line represents the lower bound of the LL ASCs half-life estimated by profile likelihood by Pasin et al.^16^ on Phase I data. The red dashed vertical line represents the newly estimated lower bound using both Phase I and II data.

The model performed well in forecasting short- and long-term humoral response. However, the increase in RMSE and the width of 95% prediction intervals, and the decrease in the percent coverage beyond 12 months (see Table 3 and Supplementary Table 1) motivated an update of the parameters using all available data from both the Phase I and Phase II trials. First, the model was modified to include laboratory effects (Battelle, Focus, or Q^2^ Solutions) in the observation model. Among the four observation models tested (see the “Methods” subsection “Update and re-estimation of the model” for more details), none was able to outperform the model without adjustment for laboratories. However, since laboratory effects reflect an observable reality, the adjusted observation model was constrained and the model including a scaling factor between the observations and the compartment Ab in natural scale, providing the lowest corrected Bayesian information criteria (BICc), was chosen. We secondly focused on the half-life of LL ASCs, $\log (2)/{\delta }_{{\rm {L}}}$. The estimation of the lower bound of the loss rate of LL ASCs *δ*_L_ was performed with a profile likelihood. Thanks to the longer follow-up available, the previous estimation of 5 years for the lower bound of the half-life of LL ASCs was updated to 15 years (Fig. 4). The method used to achieve this estimation is described in Supplementary Methods. In other words, since long-lived antibody-secreting cells are non-proliferating cells^23^, half of these antibody-secreting cells, which are produced at 7 days after the second vaccination, should persist for at least 15 years. Given this result, further estimations were performed with the parameter *δ*_L_ as fixed at the value corresponding to a lifespan of 15 years.

The application of three algorithms of covariate selection (SCM, COSSAC, and SAMBA; see the “Methods” subsection “Update and re-estimation of the model” for more details) enabled us to identify factors influencing the dynamics of the humoral response. Although methods of covariate search differ from one algorithm to another, the adjustment of the biological parameters of the model for baseline characteristics, including demographics, selected by the different methods were quite consistent. All procedures led to the selection of an effect of continent, sex, and age on antibody responses. The same covariates were identified in the model without adjustment for laboratories.

The best model estimated the effect of the continent on *ϕ*_L_ (see Table 4 presenting a summary of parameter estimations). The mean value of *ϕ*_L_ was estimated at 36.6 ELISA units/mL/days in Europe compared to 10.2 ELISA unit/mL/days in African participants. These results are in accordance with those previously obtained with Phase I trial data^16^.

**Table 4:** Model parameters estimated on Phase I participants by Pasin et al.^16^ and the new estimates obtained on pooled Phase I and Phase II data and considering adjustment for laboratory effects.

Parameter	Meaning	Phase I data		Phase I & II data	
		Mean	95% CI	Mean	95% CI
*Fixed effects*					
*δ*_Ab_	Antibody decay rate (day^−1^)	0.029	[0.027; 0.033]		
Women				0.0251	[0.0223; 0.0283]
Men				0.0353	[0.0296; 0.0421]
$\log ({{{2}}})/{{{{\delta }}}}_{{{{\rm{Ab}}}}}$	Antibody half-life (days)	24	[22; 26]		
Women				27.6	[24.5; 31.1]
Men				19.6	[16.4; 23.4]
*δ*_S_	SL ASCs decay rate (day^−1^)	0.231	[0.15; 0.36]	0.333	[0.326; 0.340]
$\log ({{{2}}})/{{{{\delta }}}}_{{{{\rm {{S}}}}}}$	SL ASCs half-life (days)	3.0	[1.9; 4.7]	2.08	[2.04; 2.13]
*δ*_L_	LL ASCs decay rate (year^−1^)	3.16 × 10^−4^	[1.46; 7.03] × 10^−4^	1.25 × 10^−4^	
$\log ({{{\bf{2}}}})/{{{{\boldsymbol{\delta }}}}}_{{{{\boldsymbol{L}}}}}$	LL ASCs half-life (years)	6.0	[2.7; 13]	15.0	
*ϕ*_S_	SL ASCs influx (EU/mL/day)	2755	[1852; 4100]		
Mean Age (31.3 years)				3057	[2418; 3865]
FC ΔAge = + 1 year^a^				0.934	[0.915; 0.954]
*ϕ*_L_	LL ASCs influx (EU/mL/day)				
African part.		16.6	[13.7; 20.1]	10.2	[9.01; 11.4]
Eur. part.		70.7	[54.0; 92.7]	36.6	[27.3; 49.2]
*α*	Scaling factor—lab effects				
*α*_focus_				1.04	[0.93; 1.16]
*α*_Q2sol_				1.00	[0.98; 1.02]
*Random effects*					
${{{{\omega }}}}_{{{{{\phi }}}}_{{{{\rm{S}}}}}}$	Sd of RE on *ϕ*_S_	0.92	[0.83; 1.01]	0.84	[0.56; 1.13]
${{{{\omega }}}}_{{{{{\phi }}}}_{{{{\rm{L}}}}}}$	Sd of RE on *ϕ*_L_	0.85	[0.78; 0.92]	0.88	[0.81; 0.96]
${{{{\omega }}}}_{{{{{\delta }}}}_{{{{\rm{Ab}}}}}}$	Sd of RE on *δ*_Ab_	0.30	[0.24; 0.36]	0.35	[0.29; 0.41]
*Error model*					
*σ*_Ab_	Sd of error model	0.10	[0.10; 0.10]	0.107	[0.101; 0.112]

*CI* confidence interval, *EU* ELISA units, *Eur.* European, *FC* fold change, *LL ASCs* long-lived antibody-secreting cells, *Part.* participants, *RE* random effects, *SL ASCs* short-lived antibody-secreting cells, *Sd* standard deviation.

^a^Represents the multiplicative factor to apply to the value of *ϕ*_S_, obtained for the mean age, for an increase in participant age of 1 year: *ϕ*_S_(Mean Age + 1 year) = *ϕ*_S_(Mean Age) × FC(ΔAge = +1). Therefore, the percentage of decrease of *ϕ*_S_ for a participant *X* years older than the mean age is given by 100×(1−FC(ΔAge = +1)^*X*^).

By adding more information with Phase II trial data, we also identified sex as another significant covariate for explaining the inter-individual variability of the decay rate of antibodies. Indeed, we estimated that antibodies have a significantly higher half-life in women (*p*-value estimated by Wald test <0.001) with an increase of the decay rate of 41% (95% confidence interval (CI): [24%; 60%]) for men as compared to women.

We also found that older age was associated with a decrease in the influx of short-lived ASCs (parameter *ϕ*_S_). For example, a 31-year-old participant (ages are assumed to be centered, see Table 1) displayed a mean value of SL ASCs influx of 3057 ELISA units/mL/days (see Table 4). Each additional year from this mean age induces a division of the resulting influx of SL ASCs by 7% (95% CI: [5%; 9%]). Therefore, for a participant 10 years older, its influx of SL ASCs will then be divided by 49% (see Table 4 footnote), corresponding to a decrease of the peak of its dynamics of 0.23 [0.22; 0.25] log_10_ EU/mL.

**Fig. 5:**
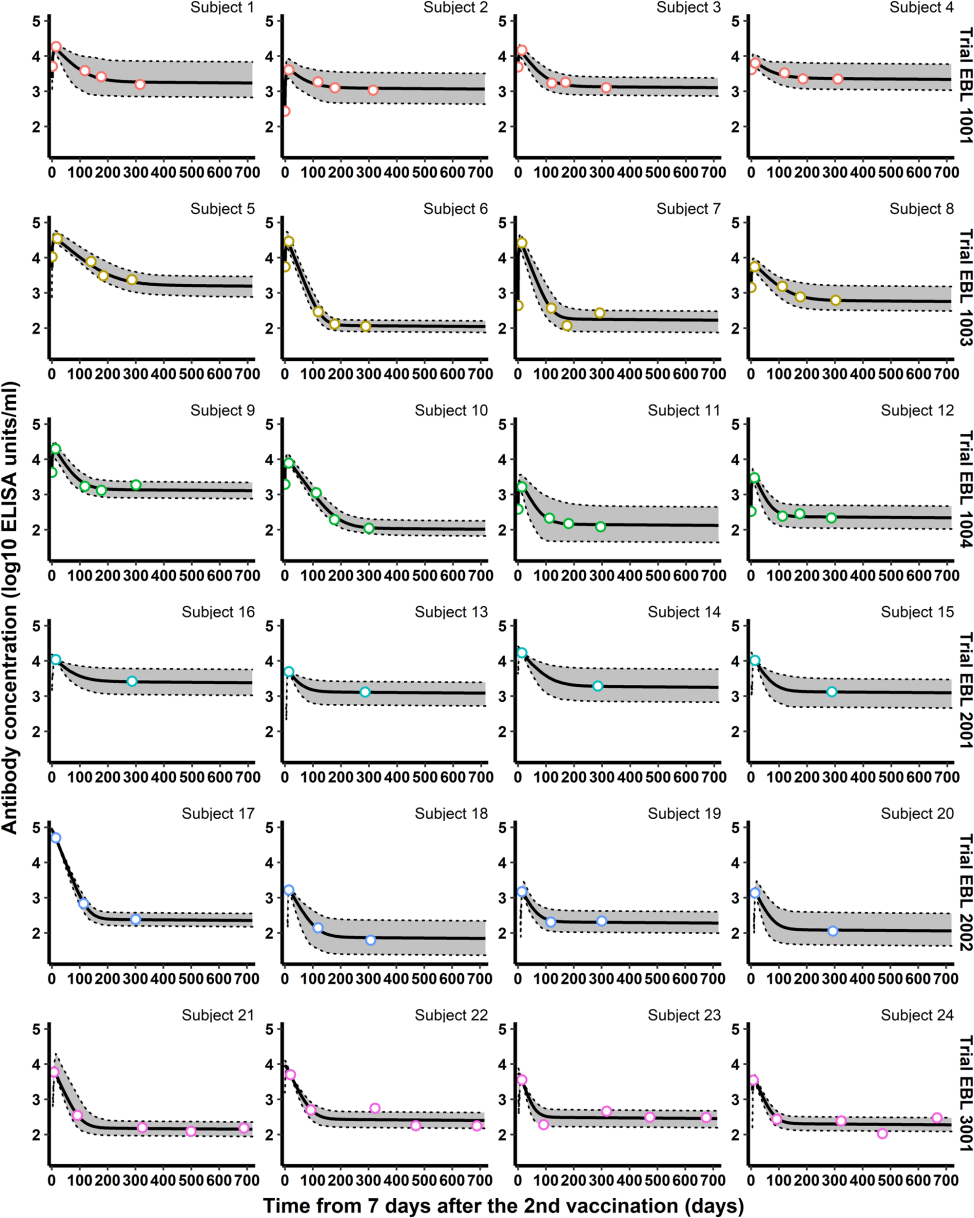
Individual antibody concentrations estimated by the model for a random sample of participants from the six clinical studies. Each subplot represents the individual antibody dynamics (in log_10_ ELISA units/mL) from 7 days after the 2nd vaccination to 2 years. Colored circles correspond to observations used to estimate the model. The thick solid lines correspond to the individual dynamics and the 95% individual confidence intervals (accounting for the uncertainty of the estimation of the individual parameters only) are delimited by the shaded areas.

Once the optimal structure was identified, we estimated the value of the parameters of the model as shown in Table 4, providing the model parameters estimated by Pasin et al.^16^ on Phase I data as well as the model parameters obtained on combined Phase I and II data. Figure 5 displays the dynamics estimated by the model, highlighting the goodness-of-fit of the data (the reader can refer to Supplementary Figs. 7 and 8 for additional results about model estimation and its goodness-of-fit).

Compared with the estimates we obtained from the Phase I data, the new estimates show a decrease in the magnitude of the *ϕ*_L_ parameter. This decrease is likely due to the significant increase in the half-life of the LL ASC from 6 to 15 years. Nevertheless, the mean *ϕ*_L_ remained four times higher in Europe than in Africa, similar to the approximation previously obtained using Phase I data^16^, where the mean *ϕ*_L_ was 4.3 times higher in Europe than in Africa. For the dynamics of SL ASCs, the parameter estimates remained quite stable, between the newly estimated model and the earlier estimates. As noted above, the information gained from longer follow-up allowed an update of the lower bound of the LL ASC decay rate. Similarly, the use of 443 additional participants improved the precision of the model parameter estimates. Indeed, the confidence intervals in the new estimates have become narrower, for each parameter *ϕ*_L_, *ϕ*_S_, or *δ*_S_, and are mostly included within the confidence intervals of the old estimates. Moreover, a comparison of the model estimates showed a slight increase in inter-individual variability for parameters *ϕ*_L_ and *δ*_Ab_ in the new model compared with the old one. The latter may be due to the use of additional data collected in a more heterogeneous population than in Phase I studies. However, adjustment of the parameter *ϕ*_S_ for the age of the participants reduced the unexplained inter-individual variability for the same parameter by 24%. Finally, it can be noticed the absence of effects of laboratory adjustment, with the two scaling factors *α*_focus_ and *α*_*Q*2Sol_ estimated as non-significantly different from 1.00, at 1.04 and 1.00, respectively.

In order to evaluate the impact of fixing the parameter *δ*_L_, we performed a model averaging analysis^24,25^, which integrates model uncertainty in the value of *δ*_L_ in the calculation of the parameter confidence intervals. Results shown in Supplementary Tables 2 and 3 indicate very stable estimation.

**Fig. 6:**
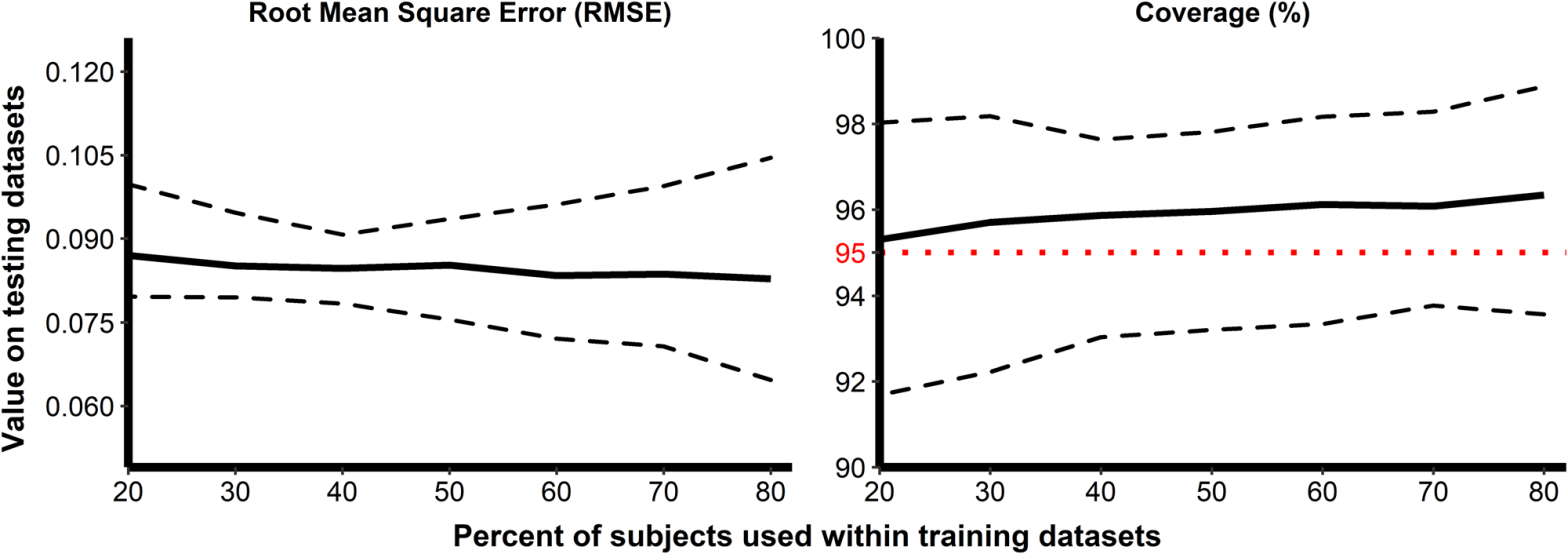
Evaluation of the ability of the model to predict unseen data using Monte-Carlo cross-validation. The predictive quality was assessed by the evaluation of two criteria: the RMSE (left side) and the percent coverage (right side). The *x*-axis corresponds to the percent of participants randomly selected for the training dataset and the *y*-axis to the value of criteria calculated on the testing dataset. One hundred replicates were performed for each train-test split percentage. Solid lines display the values of criteria and dashed lines, the 95% confidence intervals. The horizontal red dotted line on the right side displays the threshold of 95%.

We examined the ability of our model to predict the response for new participants by performing Monte Carlo cross-validation (MCCV) and using RMSE and percent coverage as quality criteria for prediction. (see the “Methods” subsection “Update and re-estimation of the model” for more details). The results of this analysis are summarized in Fig. 6, where these two criteria are displayed as functions of the percent of participants used in the training dataset. Finally, despite the wide range of percentages tested for the split of train and test, the quality of the model prediction was very stable. The mean RMSE gradually decreased from 0.0870 to 0.0828 ${\log }_{10}$ ELISA units/mL until it reached the value of 0.0843 ${\log }_{10}$ ELISA units/mL when 100% of the data were used to estimate the models. The mean percent coverage remained higher than 95% even when only 20% of participants were used to estimate the model. Consequently, the model showed reasonably good quality in predicting the humoral immune response from 7 days after the second vaccination to two years after the first vaccination.

## Discussion

With this modeling work, we evaluated the quality of long-term predictions of the mechanistic model developed by Pasin et al.^16^ which considers two populations of ASCs. We demonstrated with new data and a longer follow-up from phase 2 trials that the model, developed with a small number of participants from phase 1 trials, provides long-term predictions of the antibody response with high validity. Re-estimation of the model with a longer-term follow-up allowed us to update the value of the lower limit of the LL ASC half-life and showed that the longevity of LL plasma cells is much longer than previously estimated.

One advantage of this modeling work is the ability to distinguish the half-life of the antibodies from that of ASCs. The estimated values obtained for the half-life of the antibodies (*δ*_Ab_) were in the range of those reported in the literature of 20–50 days^26–32^. References on ASC half-lives are more difficult to figure out because these cells are circulating in various compartments (lymphoid, bone marrow) and the survival of the cells may vary greatly according to the infectious agents^33^. Hence, estimates of short-lived ASC vary from days^34^ to several weeks^3,35^. The half-life of long-lived ASCs is even more variable and reflects the variability of the antibody dynamics according to infectious agents and type of vaccines^36^. The updated estimate for the long-lived ASCs calculated in the present study is particularly encouraging with a half-life of at least 15 years. This is comparable to the half-life estimated for long-lived ASCs induced by the Hepatitis A virus vaccine^3^. Long-term humoral immunity is maintained through the memory B cells and the long-lived plasma cells^37^. These latter cells, residing preferentially in the bone marrow, produce antibodies in the absence of antigenic stimulation. Interestingly, it has been recently demonstrated that these cells are intrinsically long-lived and can maintain the secretion of antibodies without replenishment of the pool of memory B cells^38^. Specific niches in the bone marrow promote cell survival through various factors^36^.

The model was able to capture inter-individual variation of the antibody dynamics. A part of this variability was associated with the geographic region, age, and sex. The cause of the influence of geographic regions on the humoral response to vaccines is still unknown. Concomitant malaria infection is suspected to play a role in compromising the immune response^39–41^. Nevertheless, these results should be interpreted cautiously, as simple nonspecific cross-reactivity within the assays used could also be responsible for this association^40^. Chronic parasitic infections, such as schistosomiasis^42^, have also been suspected to play a role in dampening immune response to vaccination^43^. Both hypotheses are currently explored in the EBOVAC consortia. The less pronounced decline of antibodies observed in women is consistent with several reports from other vaccines, including SARS-CoV-2^44,45^, Ebola^46^, and Flu^47^. The biological mechanism behind the lower clearance of binding antibodies in women remains an area of research and may differ from vaccine to vaccine^47^. It could also be a limitation of the modeling work in identifying precisely enough which compartment is influenced by sex differences in the absence of more immunological measurements. The influence of age on the response to the vaccine is known^48^, but the characterization of its effect through the production of short-lived antibody-secreting cells is novel and requires further confirmation. Pritz et al.^49^ have noted an age-related decline in the number of plasma cells in human bone marrow. Here also, the identifiability of this effect could have been compromised because of a restricted range of participant ages because none of them were older than 65 years.

In conclusion, the dynamical model constructed from early Phase I data has demonstrated its predictive capacity, with longer follow-up and updated estimates giving promising results for the duration of the immune response. Nevertheless, the simplicity of this model significantly limits its use to fully describe the immune response induced by a multi-dose vaccine strategy. Indeed, the model focuses exclusively on the decrease in antibody concentrations following vaccination, without modeling the establishment of the immune response triggered by each injection. Thereafter, more complex versions of this model have been proposed to model the establishment, reactivation, and persistence of the humoral immune responses induced by vaccination^50,51^. In particular, these models integrate the stimulation of the immune system triggered by vaccine antigens and the role of immunological memory resulting from interactions with memory B cells and plasma cells.

The main findings obtained in this modeling work are not necessarily specific to the Ad26.ZEBOV, MVA-BN-Filo vaccine strategy. Some parameters, such as the long-lived ASCs half-life, could be found with other vaccine regimens. Nevertheless, the value and the interpretation of the parameters are dependent on the model structure and assumptions. For instance, in the modeling work conducted by Clairon et al.^51^ to model antibody dynamics induced by mRNA vaccine strategies against SARS-CoV-2, the absence of long-term persistence of antibody responses required an adaptation of the model for monophasic trajectories. In our work, the biphasic nature of the model fitted antibody trajectories perfectly, resulting in the estimation of the long-lived ASCs half-life.

The sporadic nature of Ebola outbreaks makes the identification of correlates of protection difficult^52^. Currently, neither a universal Ebola immune correlate of protection nor a binding antibody concentration threshold which would ensure a high probability of protection against EBOV, has been identified. Nevertheless, studies performed in non-human primates^14^ identified vaccine-induced binding antibody concentrations as the immune parameter most highly correlated with survival after the EBOV challenge. Due to the shorter disease course and full lethality of the Ebola disease model in non-human primates, the extrapolation of these results from animals to humans remains difficult. Consequently, no protective threshold of post-vaccination binding antibody concentration was derived for the Ad26.ZEBOV, MVA-BN-Filo vaccine.

## Methods

### Ethics statement

The Phase I UK trial protocol and study documents were approved by the UK National Research Ethics Service. The Phase I Kenya trial protocol and study documents were reviewed and approved by the local Ethics Committee and the Kenyan regulatory authority. The Phase I Uganda/Tanzania trial protocol and study documents were reviewed and approved by the Tanzanian Medical Research Coordinating Committee of the National Institute for Medical Research, the Tanzania Food and Drugs Authority, the Uganda Virus Research Institute Research and Ethics Committee, the Uganda National Council for Science and Technology, the Uganda National Drug Regulatory Authority, and the Ethics Committee of the London School of Hygiene and Tropical Medicine. The Phase II UK/France trial protocol and study documents were approved by the French National Ethics Committee (CPP Ile de France III; 3287), the French Medicine Agency (150646A-61), the UK Medicines and Healthcare Products Regulatory Agency (MHRA), and the UK National Research Ethics Service (South Central, Oxford; A 15/SC/0211). The Phase II Kenya/Uganda/Burkina Faso/Ivory Coast trial protocol and study documents were approved by local and national independent Ethics Committees and Institutional Review Boards. The Phase II Sierra Leone trial protocol and study documents were approved by The study was approved by the Sierra Leone Ethics and Scientific Review Committee, the Pharmacy Board of Sierra Leone, and the London School of Hygiene & Tropical Medicine ethics committee.

These trials were conducted in accordance with the principles of good clinical practice and the Declaration of Helsinki, and all participants gave formal, written consent before undergoing any trial-related procedure.

### Immunogenicity measurements

We considered data from six studies aiming at evaluating the safety, tolerability, and immunogenicity of two-dose vaccine regimens with Ad26.ZEBOV and MVA-BN-Filo. Ad26.ZEBOV is a monovalent, recombinant, E1/E3-deleted, replication-defective, adenovirus type 26 vector vaccine encoding Ebola virus Mayinga variant GP, produced in PER.C6 human cells and injected as a single dose of 5 × 10^10^ viral particles. MVA-BN-Filo is a recombinant, replication-defective, modified vaccinia Ankara vector vaccine encoding Mayinga variant GP, Sudan virus Gulu variant GP, Marburg virus Musoke variant GP, and Tai Forest nucleoprotein. This multivalent vaccine was produced in chicken fibroblasts and injected at a dose of 1 × 10^8^ Infectious Units (Inf. U). Three of the six studies are randomized, observer-blinded, placebo-controlled Phase I trials on healthy volunteers aged 18–50 years. These studies were performed in four countries: the United Kingdom (UK), Kenya, Tanzania and Uganda. Results of the trials were previously described by Milligan et al.^53^ and Winslow et al.^54^ for the UK (study registered at ClinicalTrials.gov, NCT02313077, and labeled EBL1001 here), Mutua et al.^55^ for Kenya (study registered at ClinicalTrials.gov, NCT02376426, and labeled EBL1003 here), and Anywaine et al.^56^ for Tanzania/Uganda (study registered at ClinicalTrials.gov, NCT02376400, and labeled EBL1004 here). In addition, we considered data from two randomized, observer-blinded placebo-controlled, parallel-group Phase II trials on healthy volunteers aged 18–65 or 75 years. These studies were performed in six countries: the UK, France, Kenya, Uganda, Burkina Faso and Ivory Coast. We refer to Pollard et al.^17^ for a detailed description of results in the European trial and to Barry et al.^18^ for the African trial (two studies registered at ClinicalTrials.gov, NCT02416453 and NCT02564523, and labeled EBL2001 and EBL2002 here respectively for the European and African studies). The last study is a combined open-label, non-randomized stage 1, and a randomized, observer-blinded, placebo-controlled stage 2 Phase II trial on healthy adults. This study conducted in Sierra Leone also aimed to evaluate the long-term immunogenicity and the humoral immune memory induced by the vaccine regimen. Results of this trial were described by Ishola et al.^19^ (study registered at ClinicalTrials.gov, NCT02509494, and labeled EBL3001 here).

In Phase I trials, participants were equally randomized into four vaccination regimens: two with MVA-BN-Filo as the first vaccination on day 1, followed by Ad26.ZEBOV on day 29 or 57, and two with Ad26.ZEBOV was the prime vaccine on day 1, followed by MVA-BN-Filo on day 29 or 57. Within each regimen, participants received either an active vaccine or placebo in a 5:1 ratio. In the study EBL2001, participants in Cohorts I–III were equally randomized into three parallel groups in which they received Ad26.ZEBOV was the first vaccine on day 1, followed by MVA-BN-Filo on day 29, 57, or 85. This first cohort was excluded from the analysis as participants were enrolled to provide data only on safety and the timing of anti-Ebola virus GP ASCs responses. Within each group, participants received active vaccines or placebo in a 14:1 or 10:3 ratio in cohorts II and III respectively. In the study EBL2002, healthy adults (Cohort I) were equally randomized into the same three parallel groups with an active vaccine: placebo ratio of 5:1. Adults HIV-infected patients (Cohort IIa) and healthy children (Cohorts IIb and III) were not included in the analysis. Finally, in the study EBL3001, participants received either Ad26.ZEBOV as first vaccination on day 1 followed by MVA-BN-Filo on day 57, or MenACWY vaccine on day 1 and placebo on day 57, with a ratio of 1:0 and 3:1 in stage 1 and 2 respectively. In this work, only participants receiving Ad26.ZEBOV as the first vaccination on day 1 and MVA-BN-Filo as the second vaccination in the protocol-defined window of 57 ± *X* days (Ad26/MVA D57; with *X* = 1 for Phase I trials and EBL2001, 3 for EBL2002 and 7 for EBL3001) were included. Based on these criteria, a total of 487 participants over all studies were included (among the 725 participants enrolled to receive Ad26/MVA D57, a total of 238 participants were excluded for not receiving their second dose (*n* = 108) or outside the protocol-defined window (*n* = 130)), 44 of whom where in Phase I studies, 71 in EBL2001, 137 in EBL2002 and 235 in EBL3001. In addition, the 168 participants receiving a placebo as a vaccine strategy were excluded.

**Fig. 7:**
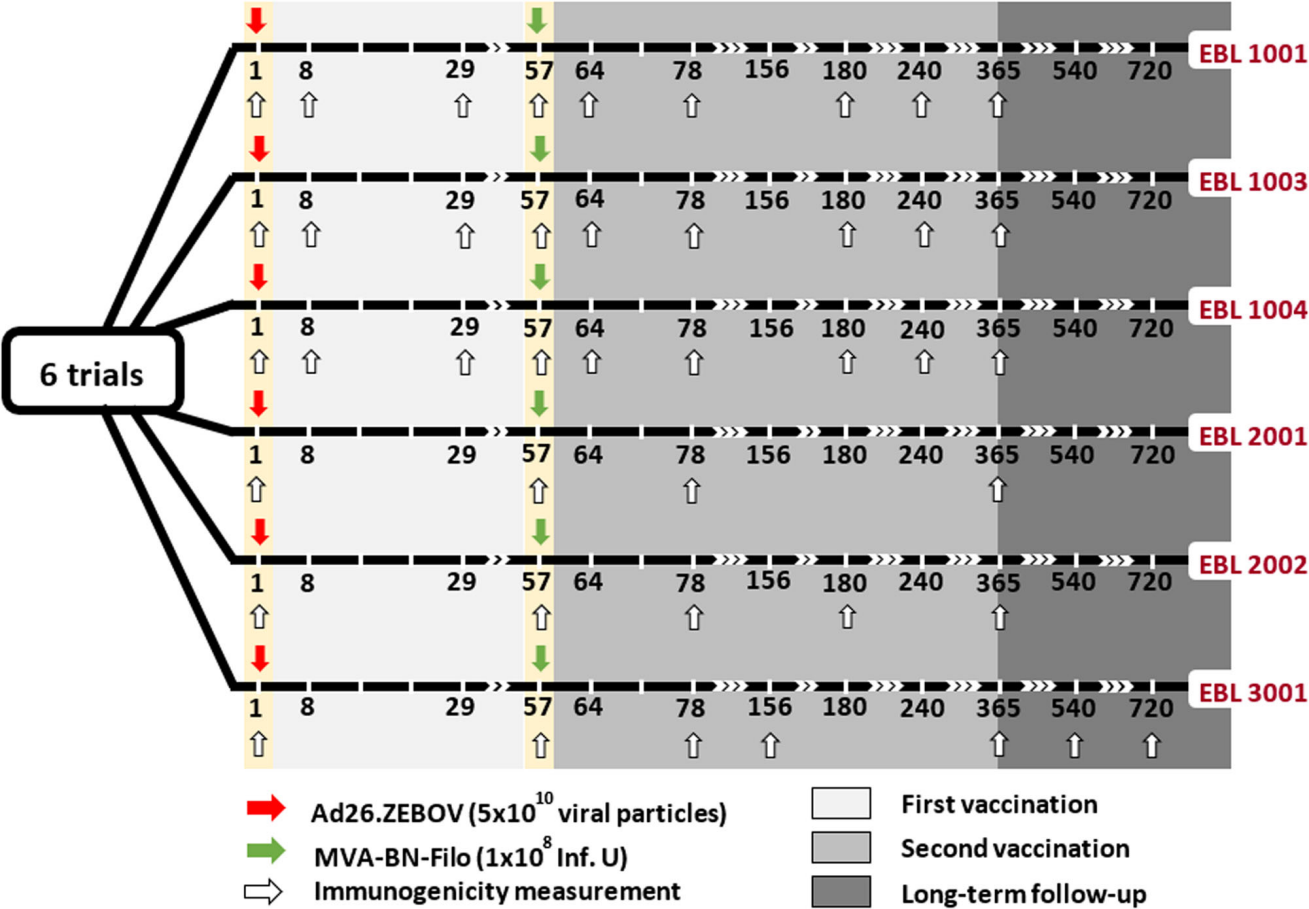
Design of EBOVAC 1 (EBL 1001, 1003, 1004, and 3001) and EBOVAC 2 (EBL 2001 and 2002) trials for participants receiving Ad26, MVA D57 as vaccine regimen. Immunogenicity measurements provide the concentration of IgG-binding antibodies against Ebola, as measured by ELISA (ELISA units/mL).

Participants were followed up to 1 year after the first vaccination in all the studies, with longitudinal immunogenicity measurements performed on blood samples. As shown in Fig. 7, for the vaccine regimen of interest, immunogenicity samples were collected in all participants immediately before the administration of the first vaccination (Ad26.ZEBOV) on day 1, before the second vaccination (MVA-BN-Filo) on day 57, then 21 days after the second dose at day 78 and 1 year after the first dose (at day 360 or 365 according to the trial). In Phase I trials, additional samples were taken at days 7, 29, 64, 180, and 240, while immunological assays were done on blood samples taken at day 180 and day 156 in EBL2002 and EBL3001 respectively. Participants enrolled in EBL3001 were additionally followed up to 2 years after the first vaccination, with blood samples collected every 6 months after the first year. We analyzed total IgG Ebola virus GP-specific binding antibody concentrations measured by an Ebola virus GP (Kikwit strain) Filovirus Animal Non-Clinical Group (FANG) ELISA assay. The FANG ELISA assays were performed at three different accredited laboratories: (a) at Battelle Biomedical Research Center (Columbus, OH, USA; hereafter referred to as Battelle) for the studies EBL1001 and EBL1004, (b) at Focus Diagnostics (San Juan Capistrano, CA, USA; hereafter referred to as Focus) for the study EBL1003, and (c) at Q^2^ Solutions Laboratory (San Juan Capistrano, CA, USA; formerly Focus Diagnostics; hereafter referred to as Q^2^ Solutions) for the studies EBL2001, EBL2002 and EBL3001. Particular attention has been paid in this work to account for a possible systematic difference in measurements induced by the distinct ELISA assays and thus between studies. Being interested in the longevity of the long-term immunity induced by the two-dose heterologous vaccine, similarly to Pasin et al.^16^, we mainly focused our analysis on immunogenicity measurements assessed after the second vaccination.

### Statistical analysis

A preliminary descriptive analysis was performed on the baseline and demographic characteristics of the 487 participants to describe and summarize the basic features of the data. Statistical differences among groups of participants were evaluated using classic *t*-tests (Welch’s *t*-test in case of unequal variance, identified by a *F*-test, and Student *t*-test otherwise) implemented in R, and *p*-values were adjusted for test multiplicity with Benjamini and Hochberg correction^20^ using the built-in R function *p.adjust*. Because of the difference in antibody concentrations measured by the distinct laboratories, comparisons of immunogenicity data between trials were not possible.

Finally, Spearman correlations between antibody concentrations measured 21 days after the second vaccination and longer-term humoral responses were evaluated by integrating adjustment for test multiplicity on *p*-values.

### Mathematical model of antibody kinetics

To analyze the humoral immune response induced by the two-dose heterologous vaccine regimen Ad26.ZEBOV, MVA-BN-Filo against Ebola virus and evaluate the long-term immunogenicity, we used a mechanistic model divided into three parts. First of all, a mathematical model based on ordinary differential equations is defined to describe the dynamics of plasma cells and antibodies^3^. As shown in Fig. 2, antibodies are assumed to be produced by two plasma cell populations differentiated by their lifespan: short- and long-lived antibody-secreting cells (ASCs). Consequently, the ordinary differential equation (ODE) system contains three compartments: the short-lived ASCs (labeled S), the long-lived ASCs (labeled L) and the antibodies (Ab). Based on the hypothesis that antibody-secreting cells peaked at day 7 post-infection/vaccination^21,22^, time was rescaled to consider only the antibody dynamics from 7 days after the second vaccination (day 64) during which plasma cells only decreased over time (*t* = time observation−64). As demonstrated by Pasin et al.^16^, the model can be written as a single equation (1).

1 $$\left\{\begin{array}{l}\frac{{\rm {dAb}}}{{\rm {d}}t}={\phi }_{{\rm {S}}}{{\rm {e}}}^{-{\delta }_{{\rm {S}}}t}+{\phi }_{{\rm {L}}}{e}^{-{\delta }_{{\rm {L}}}t}-{\delta }_{{\rm {Ab}}}{\rm {Ab}}\\ {\rm {Ab}}(t=0)={\rm {A{b}}}_{0}={\rm {A{b}}}_{D64}\end{array}\right.$$

with *δ*_S_, *δ*_L_ and *δ*_Ab_ representing the average decay rates of SL ASCs, LL ASCs and antibodies, respectively. The parameters *ϕ*_S_ and *ϕ*_L_ are, respectively, the influx of SL and LL ASCs defined as *ϕ*_S_ = *θ*_S_*S*_0_ and *ϕ*_L_ = *θ*_L_*L*_0_, where *S*_0_ = *S*(*t* = 0) = *S*_D64_ and *L*_0_ = *L*(*t* = 0) = *L*_*D*64_ are the initial conditions at 7 days after the second vaccination and *θ*_S_ and *θ*_L_ are their respective antibody production rates. The initial antibody concentration Ab_0_ is defined by the individual measure of antibody concentration at 7 days after the second vaccination. Keeping in mind that the antibody concentration can be unobserved at day 64 for some participants (see Fig. 7) while the decrease of the dynamics of ASCs is still assumed to start 7 days post-vaccination, an individual lag-time *T*_*i*_ was introduced in Eq. (1). This lag-time represents the individual time interval between day 64 and the first observation following this specific time. The equation can then be written as follows.

2 $$\left\{\begin{array}{l}\frac{{\rm {dAb}}}{{\rm {d}}t}={\phi }_{{\rm {S}}}{{\rm {e}}}^{-{\delta }_{S}(t+{T}_{i})}+{\phi }_{{\rm {L}}}{{\rm {e}}}^{-{\delta }_{{\rm {L}}}(t+{T}_{i})}-{\delta }_{{\rm {Ab}}}{\rm {Ab}}\\ {\rm {Ab}}(t=0)={\widetilde{{\rm {Ab}}}}_{0}={\rm {A{b}}}_{D64+}\end{array}\right.$$

Based on this equation, time was rescaled for each individual such that the initial condition (Ab(*t* = 0)) coincided with the first observation following day 64 (Ab_D64+_), given: time = time observation−64−*T*_*i*_. Therefore, for a participant with a first measurement at day 64 and observations at days {64, 78, 180, 240, 360}, the lag-time is null (*T*_*i*_ = 0), rescaled time points of observations are given by {0, 14, 116, 179, 296} and Ab_D64+_ is equal to measurement at day 64, Ab_*D*64_. For a participant with a first measurement at day 78 and observations at days {78, 180, 240, 360}, the lag-time *T*_*i*_ = 78−64 = 14, rescaled time points of observations are then given by {0, 102, 162, 282} and Ab_D64+_ is equal to measurement at day 78, Ab_D78_. We estimated the five following biological parameters Ψ = (*ϕ*_S_, *δ*_S_, *ϕ*_L_, *δ*_L_, *δ*_Ab_). To account for inter-individual variability, we used a statistical model on which the five model parameters are assumed to be log-transformed, to ensure their positivity. Each parameter is then described by a mixed-effects model which depends on covariates. Each individual parameter ${{{\Psi }}}_{k}^{i}$ for the participant *i* can be defined as follows, for *k* = {1, ⋯ , 5}.

3 $$\log \left({{{\Psi }}}_{k}^{i}\right)=\log ({{{\Psi }}}_{k,0})+{\beta }_{k}{Z}_{k}^{i}+{u}_{k}^{i}$$

where Ψ_0_ is the fixed effect, *Z*_*k*_ and *β*_*k*_ are, respectively, the vectors of explanatory variables and regression coefficients related to the biological parameter Ψ_*k*_, and ${u}_{k}^{i}$ is the individual random effect assumed to be normally distributed with the variance ${\omega }_{k}^{2}$. Random effects were assumed to be independent from each other. Based on results obtained in the previous work^16^, we assumed random effects on the influx parameters, *ϕ*_L_ and *ϕ*_S_, and on the decay rate of antibodies *δ*_Ab_.

For the observation model, we modeled the observed IgG binding antibody concentrations against the Kikwit glycoprotein from the six studies by the antibody ODE-compartment. We assumed an additive error model normally distributed on the log_10_ value of the antibody concentrations, with a variance ${\sigma }_{{\rm {Ab}}}^{2}$. The antibody concentration for patient *i* at the *j*th time is given by

4 $$Y({t}_{ij})={\log }_{10}\left[{\rm {Ab}}({{{\Psi }}}^{i},{t}_{ij})\right]+{\varepsilon }_{ij}\quad {\varepsilon }_{ij} \sim {{{\mathcal{N}}}}(0,{\sigma }_{Ab}^{2})$$

### Model estimation

Mathematical and practical identifiability has been assessed in previous work^16^. Thus the parameter *δ*_L_ was estimated by profile likelihood^57^ which consists of defining a grid of values for the parameter, sequentially setting the parameter *δ*_L_ at one of those different values, and estimating the model by maximizing the log-likelihood, given that the value of *δ*_L_. The resulting profile shows the maximum possible log-likelihood for each value of *δ*_L_ and has its maximum at the maximum likelihood estimate ${\hat{\delta }}_{{\rm {L}}}$. Other parameters were estimated by a population approach in which the model estimation relies on the estimation of the vector of population parameters including the fixed effects (**Ψ**_**0**_), the regression coefficients (***β***), the standard deviation of random effects (***ω***) and the standard deviation of the error model (*σ*_Ab_). Model estimation was performed by the Monolix ®software versions 2019R1 and 2019R2. This software uses the Stochastic Approximation Expectation-Maximization (SAEM) algorithm^58,59^ to estimate the population parameters with likelihood computed by importance sampling^60^ and the Fisher information matrix calculated by stochastic approximation. Once population parameters are re-estimated, individual parameters are computed as empirical Bayes estimates (EBEs) representing the most likely values of the individual parameters, given individual data and population parameters. EBEs are calculated as the mode of the conditional parameter distribution by Markov-Chain Monte-Carlo (MCMC) procedure^61^ using the Metropolis–Hasting algorithm^62^ to compute the conditional distribution and the Nelder–Mead Simplex algorithm^63^ to maximize it.

### Evaluation of the model quality of prediction

The mechanistic model described by Eqs. (2)–(4), initially estimated on Phase I data by Pasin et al.^16^, was validated on data from the six trials according to its quality of prediction. To this end, a two-step approach was applied: first, the robustness of the model was assessed by evaluating its ability to predict antibody dynamics from 7 days post-second vaccination to the peak of the dynamics (i.e., the first local maximum) for all Phase I and Phase II participants. Then, the ability of the model to forecast short-term (i.e., from the peak to 1 year after the first vaccination) and long-term antibody concentration (i.e., beyond 1 year following the first vaccination) was evaluated. Because validation of the mechanistic model estimated on Phase I data is sought here, no modification of the observation model defined in Eq. (4) was considered here to account for possible laboratory-induced effects in the measurement of antibody concentrations.

To investigate the robustness of the model initially estimated on Phase I data, only data restricted to the first year following the first vaccination were used to stay in the scope of applicability of the model (see Table 2 for a detailed description of the number of observations available at each time point). Consequently, for each participant, the peak of its dynamics was sought during the first year (see Table 3 for a detailed description of individual times of peak in each trial). Assuming fixed effects and regression coefficients of the population parameters (Ψ_*k*,0_ and *β*_*k*_, ∀ *k* ∈ {1, ⋯ , 5}), distribution of random effects (*ω*_*k*_, ∀ *k* ∈ {1, ⋯ , 5}), as well as the standard deviation of the error model (*σ*_Ab_) as fixed to previously obtained values, we evaluated individual parameters for the 487 participants, via the variables ${u}_{k}^{i}$, using the empirical Bayes estimates (EBEs) approach implemented in Monolix. As shown in Table 4, we fixed the decay rate of antibodies (*δ*_Ab_), SL ASCs (*δ*_S_) and LL ASCs (*δ*_L_) at values corresponding to half-lives of 24 days, 3 days and 6 years, respectively. The parameter *ϕ*_S_ was fixed at 2755 ELISA units/mL/day while *ϕ*_L_ was fixed at 16.6 ELISA units/mL/day for African participants and 70.7 ELISA units/mL/day for Europeans. East and West African participants were assumed to share the same value of LL ASCs influx. Finally, standard deviations of the inter-individual variability on the three parameters *ϕ*_S_, *ϕ*_S_ and *δ*_Ab_ were chosen as ${\omega }_{{\phi }_{{\rm {S}}}}=0.92,{\omega }_{{\phi }_{{\rm {L}}}}=0.85$ and ${\omega }_{{\delta }_{{\rm {Ab}}}}=0.30$. The parameter *σ*_Ab_ was fixed at 0.10 (see ref. ^16^ or Table 4). To stay consistent with the model built on Phase I data, we included an adjustment for geographic region in the statistical model (binary variable equal to 0 in Africa and 1 in Europe) on *ϕ*_L_, as shown in the following equation:

5 $$\log ({\phi }_{{\rm {L}}}^{i})=\log ({\phi }_{{\rm {L}},0})+{\beta }_{{\phi }_{{\rm {L}},{\rm {Eur}}}}\times {{\mathbb{1}}}_{i\in {\rm {Eur}}}+{u}_{{\phi }_{{\rm {L}}}}^{i}$$

For each individual, the 95% prediction interval^64^ of the antibody dynamics was calculated and the percent coverage, defined as the percent of observations falling within the prediction interval, was assessed. Through these results, we highlighted the ability of the model to predict the very first antibody concentration measurements from 7 days post-second vaccination. Once these predictions were validated, individual parameters estimated on the early phase of the follow-up were used in the second step to quantify both the short- and long-term forecast skills of the model. To this end, we used the model to make individual predictions of antibody concentration between the peak and 2 years after the first vaccination. Predictions were then compared to observations and the percent of observations falling within the 95% individual prediction intervals was quantified.

Thereafter, the two-step approach was also applied for evaluating: first, the ability of the model to predict antibody dynamics from 7 days post-second vaccination to 1 year after the first vaccination (instead of the peak), and second, its ability to forecast antibody concentration beyond 1 year. This additional analysis was performed to identify whether the estimation of individual parameters on a longer follow-up can improve long-term predictions.

*K*-means clustering for longitudinal data^65^ was performed to identify distinct trajectories of the dynamics of the humoral response. Using the kml R package^66^, trajectories of antibody concentration from 7 days after the second vaccination to 2 years after the first vaccination were sequentially clustered into two and more clusters. Thereafter, we evaluated the percent coverage and the RMSE to investigate potential differences in prediction abilities according to underlying trajectories for each resulting partition.

### Update and re-estimation of the model

Once the quality of prediction of the mechanistic model was evaluated, an update of the model was performed in order to improve biological knowledge about the longevity of the long-term immune response induced by the two-dose heterologous vaccine regimen, Ad26.ZEBOV, MVA-BN-Filo. The low number of participants included in the three Phase I trials (177 participants, of whom only 44 received the Ad26/MVA D57 vaccine regimen) as well as the short-term follow-up of their immune response up to 1 year after the first vaccination tended to limit the precision of the estimation of the model parameters in the work conducted only on Phase I trials. Despite the validation of the model according to its quality of prediction on additional data coming from the three Phase II trials (EBL 2001, 2002 and 3001), a re-estimation of the model using antibody dynamics from the 487 participants was performed to enhance and reinforce our understanding of the underlying biological processes leading to the long-term immunity following vaccination against Ebola. To account for the difference in measurements induced by the three distinct ELISA assays performed at Battelle, Focus and Q^2^ Solutions laboratories, we assumed in the observation model an adjustment for laboratory effects, as shown on the following equation:

6 $$\begin{array}{lll}Y({t}_{ij})\,=\,{\log }_{10}\left[\alpha \times {\rm {Ab}}({{{\Psi }}}^{i},{t}_{ij})\right]+{\varepsilon }_{ij}\quad {\varepsilon }_{ij} \sim {{{\mathcal{N}}}}(0,{\sigma }_{{\rm {Ab}}}^{2})\\ \qquad\alpha \,=\,\left\{\begin{array}{ll}1\,\qquad{{\mbox{if}}}\,\,i\in \,\,{{\mbox{Battelle}}}\,\\ {\alpha }_{{\rm {focus}}}\,\,{{\mbox{if}}}\,\,i\in \,\,{{\mbox{Focus}}}\,\\ {\alpha }_{{\rm {Q2sol}}}\,\,{{\mbox{if}}}\,\,i\in \,{{{\mbox{Q}}}}^{2}\,\,{{\mbox{Solutions}}}\,\end{array}\right.\end{array}$$

with *α* representing the proportional scaling factor (in natural scale) between the three laboratories, considering Battelle as the reference, and where the two parameters *α*_focus_ and *α*_Q2sol_ are estimated with the five other biological parameters (Ψ). To ensure their positivity, both of them are assumed to be log-transformed. Further investigations with other link-functions between *Y* and Ab were conducted to model laboratory effects, such as a proportional relationship in the log10 scale, or with more complex functions like nonlinear sigmoid functions applied either in the natural or log10 scale. The function leading to the best model (i.e., lowest BICc value) before any covariate adjustment was kept. We also tested whether the accuracy of the three assays differed using different measurement error models. However, this modeling did not improve the fit and was therefore not retained (result not shown).

Participants from Phase I clinical studies, being monitored only during the first year following the first vaccination, provided information only on the early phase of the humoral response. In particular, the lack of information on long-term immunity made the estimation of the decay rate of the long-lived ASCs difficult. Long-lived ASCs are persistent plasma cells with a lifespan ranging from several months to the end of an individual’s lifetime^23,67–69^, therefore only an approximation of the lower bound of the confidence interval of their half-life ($\log (2)/{\delta }_{{\rm {L}}}$) was possible. Using additional data from Phase II studies and, in particular, the humoral response measurements beyond 1 year, we performed a profile likelihood to identify whether enough information was available to precisely estimate the parameter *δ*_L_. Considering the statistical model found by Pasin et al.^16^, the model was estimated for multiple values of LL ASCs half-life ranging from 1 to 40 years. The profile likelihood was then drawn by maximizing the log-likelihood, computed by importance sampling^60^, for each of those related models.

As a first estimation, a sequential Bayesian estimation was envisaged, that is using information provided by Phase I studies only through informative prior distribution for parameters. Maximum a posteriori (MAP) estimates, corresponding to a penalized maximum likelihood estimation^70^, should then be obtained using humoral responses from only the 443 Phase II participants. However, the difference in sampling between Phase I and II studies, in particular the absence of data from 7 to 21 days after the second vaccination for Phase II participants (see Table 2), made estimation of the model difficult. The lack of information at the early stage of the dynamics induced practical identifiability issues for the parameters *δ*_S_ and *ϕ*_S_. To tackle this difficulty, all data were used to update the model. Random effects found on Phase I trials were kept, considering inter-individual variability on the parameter *δ*_Ab_ as well as on the ASCs influx, *ϕ*_L_ and *ϕ*_S_.

The statistical model was updated by performing a covariate selection. We applied the classic stepwise covariate modeling (SCM) algorithm^71,72^ which is a stepwise procedure with a forward selection followed by a backward elimination. In the forward selection, each parameter–covariate relationship is tested in turn and the relationship improving the model criteria (a corrected version of the Bayesian information criterion, BICc) the most is kept (the lower the better). Then the addition of a second covariate is tested. In the backward elimination, the removal of each parameter-covariate relationship selected in the first step is tested in an univariate manner. To verify the robustness of the results, two other algorithms of covariate selection in non-linear mixed effects models were performed, using BICc as model selection criteria: (1) the conditional sampling use for a stepwise approach based on correlation tests (COSSAC)^72^, and (2) the stochastic approximation for model building algorithm (SAMBA)^73^. The three algorithms were independently applied on an initial model without any covariates and tested the addition of the seven following potential covariates: Sex (=0 for women and =1 for men), Age, Weight, BMI, Continent (=0 for Africa and =1 for Europe), Region (=0 for East Africa, =1 for West Africa and =2 for Europe) and EBL3001 (=1 for participants from EBL3001 and 0 otherwise). Covariates such as Age, BMI, and Weight were centered around the mean value of the studied population (see Table 1). The parameter *δ*_L_, facing some identifiability issues due to the lack of measurements beyond two years, was removed from the covariate selection procedure. Based on their definition, the parameters *α*_focus_ and *α*_*Q*2sol_ were also excluded from this selection. The statistical significance of selected covariates was then evaluated using a Wald test. EBL3001 was the only study that had a follow-up beyond 1 year after the first vaccination and which was conducted in a single country (Sierra Leone). Therefore, the robustness of the results was analyzed to verify the short-term relevance of the selected covariates. To this end, the same procedure was performed on the model already adjusted for the selected covariates but considering only data up to 1 year after the first vaccination. At the end of the covariate selection procedure, an optimal model was obtained with the following statistical model (see section “Results” subsection “Additional insight on longevity of the humoral immune response” for more details).

7 $$\left\{\begin{array}{l}\log ({\phi }_{{\rm {L}}}^{i})=\log ({\phi }_{{\rm {L}},0})+{\beta }_{{\phi }_{{\rm {L,Eur}}}}\times {{\mathbb{1}}}_{i\in {\rm {Eur}}}+{u}_{{\phi }_{{\rm {L}}}}^{i}\\ \log ({\phi }_{{\rm {S}}}^{i})=\log ({\phi }_{{\rm {S}},0})+{\beta }_{{\phi }_{{\rm {S,Age}}}}\times \left({\rm {Ag{e}}}_{i}-\overline{{\rm {Age}}}\right)+{u}_{{\phi }_{{\rm {S}}}}^{i}\\ \log ({\delta }_{{\rm {Ab}}}^{i})=\log ({\delta }_{{\rm {Ab}},0})+{\beta }_{{\delta }_{{\rm {Ab,Men}}}}\times {{\mathbb{1}}}_{i\in {\rm {Men}}}+{u}_{{\delta }_{{\rm {Ab}}}}^{i}\end{array}\right.$$

where Age_*i*_ and $\overline{{\rm {Age}}}$ are, respectively, the age of the participant *i* and the average age of the participants and with ${u}_{{\phi }_{{\rm {L}}}}^{i} \sim {{{\mathcal{N}}}}(0,{\omega }_{{\phi }_{{\rm {L}}}}^{2}),{u}_{{\phi }_{\rm {{S}}}}^{i} \sim {{{\mathcal{N}}}}(0,{\omega }_{{\phi }_{{\rm {S}}}}^{2})$ and ${u}_{{\delta }_{{\rm {Ab}}}}^{i} \sim {{{\mathcal{N}}}}(0,{\omega }_{{\phi }_{{\rm {S}}}}^{2})$. Once the optimal model selected, its goodness of fit was checked and the robustness of the convergence of the estimation was assessed by using the convergence assessment tool implemented in Monolix which evaluated the robustness of the SAEM algorithm for numerous initial conditions.

The predictive quality of the newly estimated model was assessed by performing a Monte-Carlo cross-validation^74^. Participants from the overall dataset were randomly split into a training and a testing dataset, given a particular train-test split percentage. We ensure that the same ratio of participants in each trial was maintained within each of the two sub-datasets. Once the model was fitted on training data, EBEs resulting from this model were evaluated on test data, followed by the prediction of the individual antibody dynamics. Two criteria were then calculated on the testing dataset to estimate how accurately the predictive model performs: the percent coverage (the higher the better) and the RMSE (the lower the better). For each of the seven train-test split percentages {20%, 30%, 40%, 50%, 60%, 70%, 80%}, the procedure was replicated 1000 times.

### Reporting summary

Further information on research design is available in the Nature Research Reporting Summary linked to this article.

### Supplementary information

Supplementary material

Reporting Summary

## Data Availability

Janssen has an agreement with the Yale Open Data Access (YODA) Project to serve as the independent review panel for the evaluation of requests for clinical study reports and participant-level data from investigators and physicians for scientific research that will advance medical knowledge and public health. Data will be made available following publication and approval by YODA of any formal requests with a defined analysis plan. For more information on this process or to make a request, please visit the Yoda Project site at http://yoda.yale.edu. The data sharing policy of Janssen Pharmaceutical Companies of Johnson & Johnson is available at https://www.janssen.com/clinical-trials/transparency.

## Code availability

The original code (mlxtran models and R codes) developed in this work is available and free-of-cost on github (Inria SISTM Team) at the following link: https://github.com/sistm/ModelingEbola.git.
