# Supplementary material for: Prediction of long-term humoral response induced by the two-dose heterologous Ad26.ZEBOV, MVA-BN-Filo vaccine against Ebola
Source: NPJ Vaccines. 2023 Nov 8;8:174. doi: 10.1038/s41541-023-00767-y (PMC10632397; doi:10.1038/s41541-023-00767-y)
Supplement: Supplementary file 1 — Supplementary material [file 41541_2023_767_MOESM1_ESM.pdf]

Prediction of long-term humoral response induced by the two-dose heterologous Ad26.ZEBOV, MVA-BN-Filo vaccine against Ebola

Marie Alexandre[1,2], Mélanie Prague[1,2,†], Chelsea McLean[3], Viki Bockstal[3,‡], Macaya Douoguih[3], Rodolphe Thiébaut[1,2,†,*] for the EBOVAC 1 and EBOVAC 2 Consortia

[1] Bordeaux University, Department of Public Health, Inserm UMR 1219 Bordeaux Population Health Research Center, Inria SISTM ; Bordeaux, France
[2] Vaccine Research Institute; Créteil, France
[3] Janssen Vaccines and Prevention, Leiden, the Netherlands
‡ Current address: ExeVir, Ghent, Belgium.
† These authors contributed equally to this work.

***Corresponding Author:** Rodolphe Thiébaut, rodolphe.thiebaut@u-bordeaux.fr
Address: Bordeaux University, Department of Public Health, Bordeaux, France.

SUPPLEMENTARY MATERIAL

Supplementary Methods

In case of identifiability issues, a classic approach applied to improve model identifiability consists in fixing the value of poorly identifiable parameters to plausible biological values. As performed in the main analysis with the parameter of the decay rate of long-lived ASCs (δ_L), a profile likelihood can be done to identify those maximizing the log-likelihood of the model. When used to fix the value of the poorly identifiable parameter, the profile likelihood approach is equivalent to performing model selection among a set of model candidates that are differentiated by the values of this parameter and using log-likelihood as selection criteria. However, by focusing on a single value, this approach totally ignores the model selection uncertainty (1, 2). Model averaging is a simple alternative approach that can be used to deal with model uncertainty by averaging model predictions or parameter estimates of all candidate models according to their consistency with data (3).
In this work, we are interested in quantifying the impact of the uncertainty of the parameter δ_L (i.e, the decay rate of long-lived ASCs) on the model estimation. Consequently, we aim at deriving estimators of model parameters integrating this uncertainty.

Description of the model averaging approach

Definition of candidate models

Let assume a set of M models that can be used to describe our data. We note Y_{ijm} the $\log_{10}$ antibody concentration for participant i at time t_j provided by the mth candidate model, as follows:

$$Y_{ijm} = Y_m(t_{ij}) = \log_{10}\left[\alpha_m \times Ab_m\left(\Psi^i_m, t_{ij}\right)\right] + \varepsilon_{ijm} \qquad \varepsilon_{ijm} \sim \mathcal{N}(0, \sigma^2_{Ab,m}) \quad [1]$$

with Ab_m being the function describing the antibody concentration in the model m, $\boldsymbol{\Psi^i_m}$ and α_m being the vector of individual parameters and the proportional laboratory scaling factor under model m ($\alpha_m \in \boldsymbol{\Psi^i_m}$), respectively, and ε_{ijm} the residual error assumed to be normally distributed with a variance $\sigma^2_{Ab,m}$. As described in the main article (see equation (3)), each individual parameter of each candidate model m, $\Psi^i_{k,m}$, is assumed log-normally distributed with a fixed effect $\Psi_{k,m,0}$, a vector of covariates $\boldsymbol{\beta_{k,m}}$, and individual random effect, $u^i_{k,m}$, assumed to be independent and normally distributed with a variance $\omega^2_{k,m}$.

$$\log(\Psi^i_{k,m}) = \log(\Psi_{k,m,0}) + \beta_{k,m} Z^i_{k,m} + u^i_{k,m} \quad [2]$$

For each candidate model m, we can then define the vector of population parameters $\boldsymbol{\Theta_m} = \{\boldsymbol{\Psi_{m,0}}, \boldsymbol{\beta_m}, \boldsymbol{\Omega_m}, \sigma_{Ab,m}\}$, where $\boldsymbol{\Psi_{m,0}} = (\Psi_{1,m,0}, \cdots, \Psi_{K,m,0})^T$ is the vector of fixed effects, $\boldsymbol{\beta_m} = (\boldsymbol{\beta_{1,m}}, \cdots, \boldsymbol{\beta_{K,m}})$ is the matrix of covariates, and $\boldsymbol{\Omega_m} = \text{diag}((\omega^2_{1,m}, \cdots, \omega^2_{K,m}))$ is the diagonal variance-covariance matrix of random effects.

Model averaging estimation and weight choice

Once each of the M candidate models estimated, we can note $\boldsymbol{\hat{\Theta}_m}$ the maximum likelihood estimator of the population parameters $\boldsymbol{\Theta_m}$ provided by the mth model. Let note $\boldsymbol{\lambda} = (\lambda_1, \cdots \lambda_M)^T$ be the weight vector belonging to the set $\Lambda = \{\boldsymbol{\lambda} \in [0,1]^M, \sum_{m=1}^M \lambda_m = 1\}$. The model averaging estimator is then defined as

$$\boldsymbol{\hat{\Theta}}_{\text{MA}} = \sum_{m=1}^{M} \lambda_m \boldsymbol{\hat{\Theta}_m} \quad [3]$$

For each parameter population parameter $\theta \in \boldsymbol{\Theta}$, we can then derive a simple estimator of the unconditional variance for its model averaging estimator, $\hat{\theta}_{\text{MA}}$ (4):

$$\hat{\text{Var}}(\hat{\theta}_{\text{MA}}) = \sum_{m=1}^{M} \lambda_m \times \left[\hat{\text{Var}}(\hat{\theta}_m) + \left(\hat{\theta}_m - \hat{\theta}_{\text{MA}}\right)^2\right] \quad [4]$$

where $\hat{\text{Var}}(\hat{\theta}_m)$ denotes the conditional sampling variance (also referred as intra-model variance), and $(\hat{\theta}_m - \hat{\theta}_{\text{MA}})^2$ denotes the variance component for model selection uncertainty (also referred as inter-model variance). In this approach, we chose the classic smoothed AIC criterion (1) to attribute a weight to each candidate model. Hence, the model averaging weight of the mth model, λ_m, is defined as follows

$$\lambda_m = \frac{e^{\frac{-\Delta\text{AIC}_m}{2}}}{\sum_{p=1}^{M} e^{\frac{-\Delta\text{AIC}_p}{2}}} \quad [5]$$

where $\Delta\text{AIC}_m = \text{AIC}_m - \text{AIC}_{\text{min}}$ with $\text{AIC}_m = -2LL(\hat{\Theta}_m) + 2|\hat{\Theta}_m|$ and AIC_{min} being the lowest AIC value among the M candidate models.

Identification of long-lived ASCs half-life by model selection

The profile likelihood performed in the main analysis to identify optimal values for the parameter δ_L allowed to easily see a significant increase of the value of the lower bound of the half-life of long-lived ASCs. However, its flat profile made the choice of this lower bound difficult. To choose a value that is statistically meaningful, among all these biologically relevant values, we combined the profile likelihood with the rule defined by Burnham and Anderson (1) for ranking and comparing candidate models using Akaike Information Criterion (AIC). In particular, this rule stipulate that, in comparison with the optimal model showing the smallest AIC value, a model m such as (1) $\Delta\text{AIC}_m \leqslant 2$ has substantial support, (2) $4 \leqslant \Delta\text{AIC}_m \leqslant 7$ has less support but still non-negligible, and (3) $\Delta\text{AIC}_m \geqslant 10$ has no support to describe data. In that respect and our interest being to identify the lower bound of the long-lived ASCs half-life, we selected among all models involved in the profile likelihood the one with the lowest value of long-lived ASCs half-life and such that $\Delta\text{AIC}_m \leqslant 7$. This allowed us to found the lower bound of 15 years.

Application of the model averaging approach

Definition of candidate models

For sake of clarity and simplicity, we applied the model averaging approach only on the final model identified in the re-estimation step presented in the main article. As described previously, we proposed this approach as an alternative to model selection based on profile likelihood for the parameter δ_L. Consequently, the set of candidate models includes only models similar to the optimal model, (i.e., with Continent, Age and Sex covariates on parameters ϕ_L, ϕ_S and δ_{Ab}, respectively, and with the observation model adjusted for laboratory effects), whose the parameter δ_L was fixed at different values.

First, similarly to the profile likelihood performed in the main analysis, we estimated models with long-lived ASCs half-life ($\log(2)/\delta_L$) ranging from 1 to 40 years. Then, in order to reduce the number of models to average, we used the AIC-based rule defined by Burnham et al. aforementioned. Only models with a difference of AIC lower than 7 points, compared to the lowest one, were considered as candidate models (see Supplementary Figure 9), corresponding to a total of 25 models over 39.

Model averaging estimates of population parameters

Once all candidate models estimated, the equation [5] was used to calculate model averaging weights. As shown in Supplementary Figure 10, weights are quite well distributed over the 25 models. However, it is important to note that, even if we included in the set of candidate models those having a Δ AIC lower than 7 points, the formula describing model averaging weights has been defined to allocate the majority of weights among models having a Δ AIC lower than 2 points. This specificity explains why higher weights were allocated to models with the value of long-lived ASCs half-life ranging from 29 to 40 years (see Supplementary Figure 9 for Δ AIC values).

Considering population parameters estimates obtained for each model, whose distributions for parameters δ_{Ab}, δ_S, ϕ_S and Φ_L are given in Supplementary Table 2, we were able to calculate the model averaging estimators and their unconditional distributions for all population parameters. In the Supplementary Table 3, we compared the

population parameter estimates obtained in the main article ,where model selection has been performed on the value of the parameter δ_L, to those obtained by model averaging. The closeness of the estimates obtained by the two approaches highlights the lack of information contained in data to evaluate the half-life of long-lived ASCs. Nevertheless, as expected, the inclusion of uncertainty related to the value of the parameter δ_L leads to a slight increase of the confidence intervals of population parameters obtained by model averaging. These results allow, in particular, to identify Φ_S as the parameter the most sensitive to the value of the poorly identifiable parameter δ_L even though this sensitivity remains weak.

Supplementary Figures

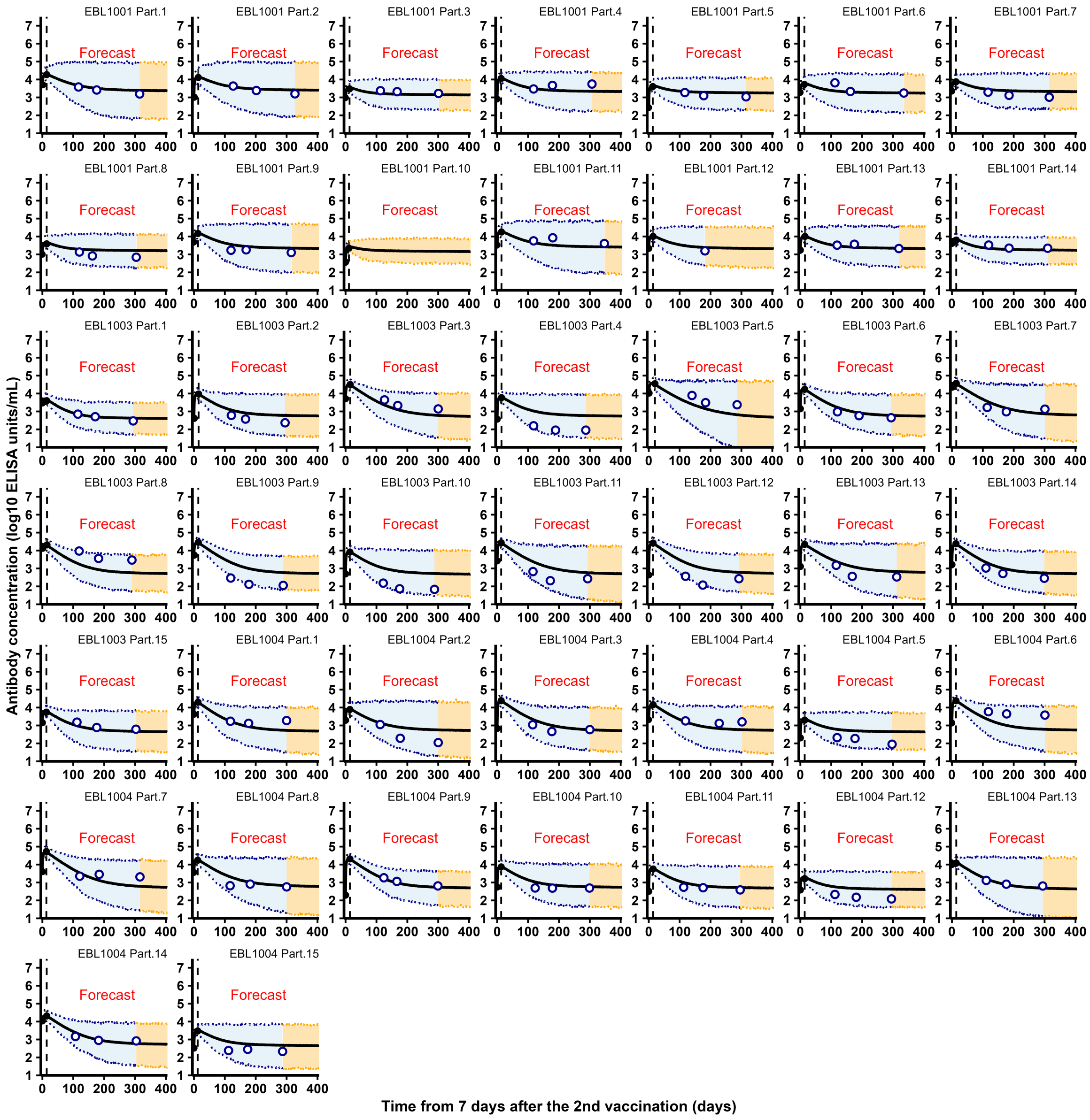

Supplementary Figure 1 – Individual antibody concentrations predicted by the model estimated on phase I data and EBEs evaluated using data restricted to the peak, for Phase I participants. Each subplot represents the individual antibody dynamics (in log10 ELISA units/mL) from 7 days after the 2nd vaccination. For each participant, the vertical dashed line represents the time limit (individual peak of dynamics) between the predictions (on the left) and the forecasts (short-term in blue and long-term in orange). Plain dots correspond to observations used to evaluate individual parameters while circles are observations not used in parameter estimation. Shaded areas correspond to 95% individual prediction intervals (accounting for the uncertainty on the individual parameter estimation and the measurement error) and the solid lines correspond to the prediction of the model.

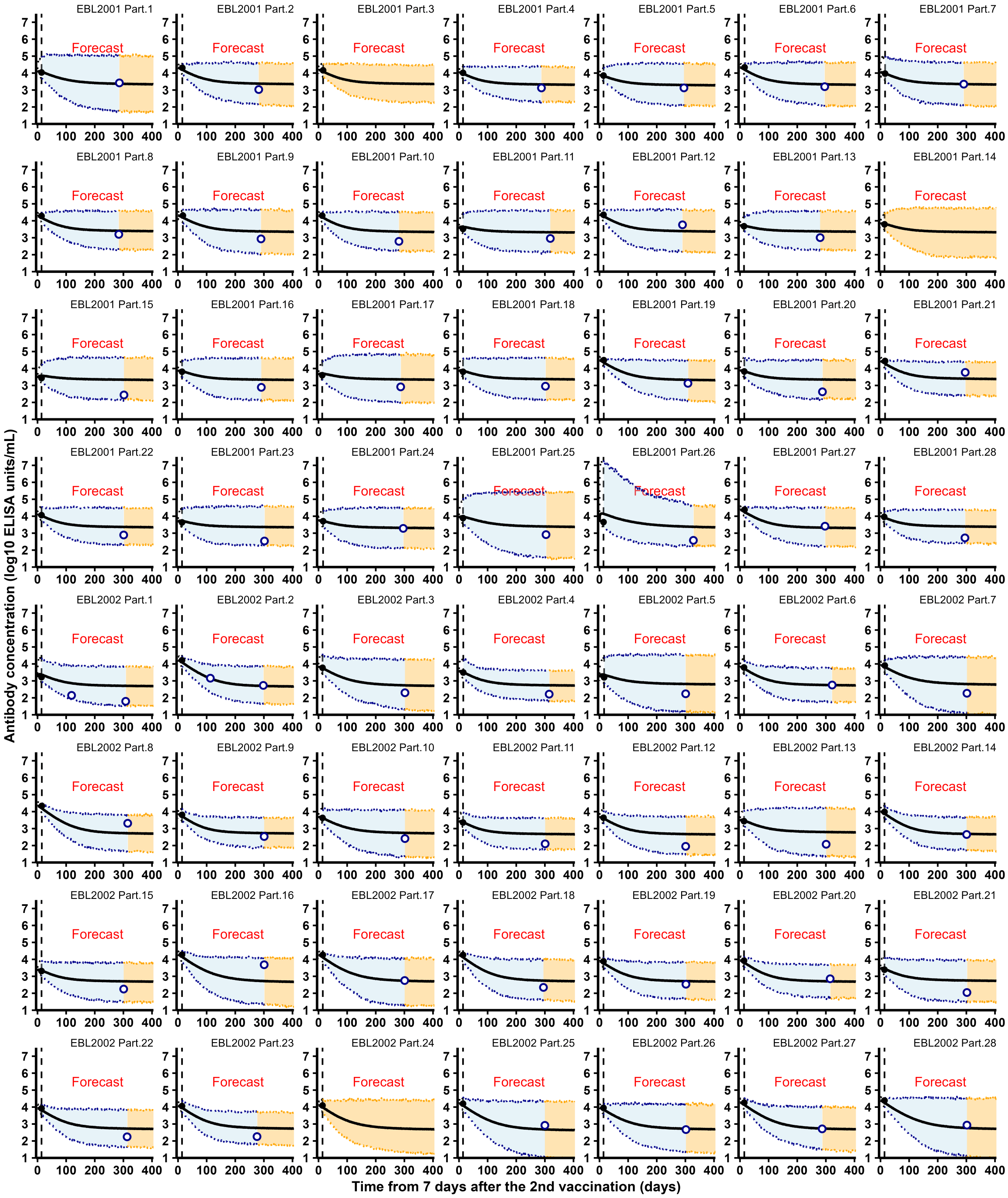

Supplementary Figure 2 – **Individual antibody concentrations predicted by the model estimated on phase I data and EBEs evaluated using data restricted to the peak, for random sample of participants from EBL2001 and EBL2002**. Each subplot represents the individual antibody dynamics (in log10 ELISA units/mL) from 7 days after the 2nd vaccination. For each participant, the vertical dashed line represents the time limit (individual peak of dynamics) between the predictions (on the left) and the forecasts (short-term in blue and long-term in orange). Plain dots correspond to observations used to evaluate individual parameters while circles are observations not used in parameter estimation. Shaded areas correspond to 95% individual prediction intervals (accounting for the uncertainty on the individual parameter estimation and the measurement error) and the solid lines correspond to the prediction of the model.

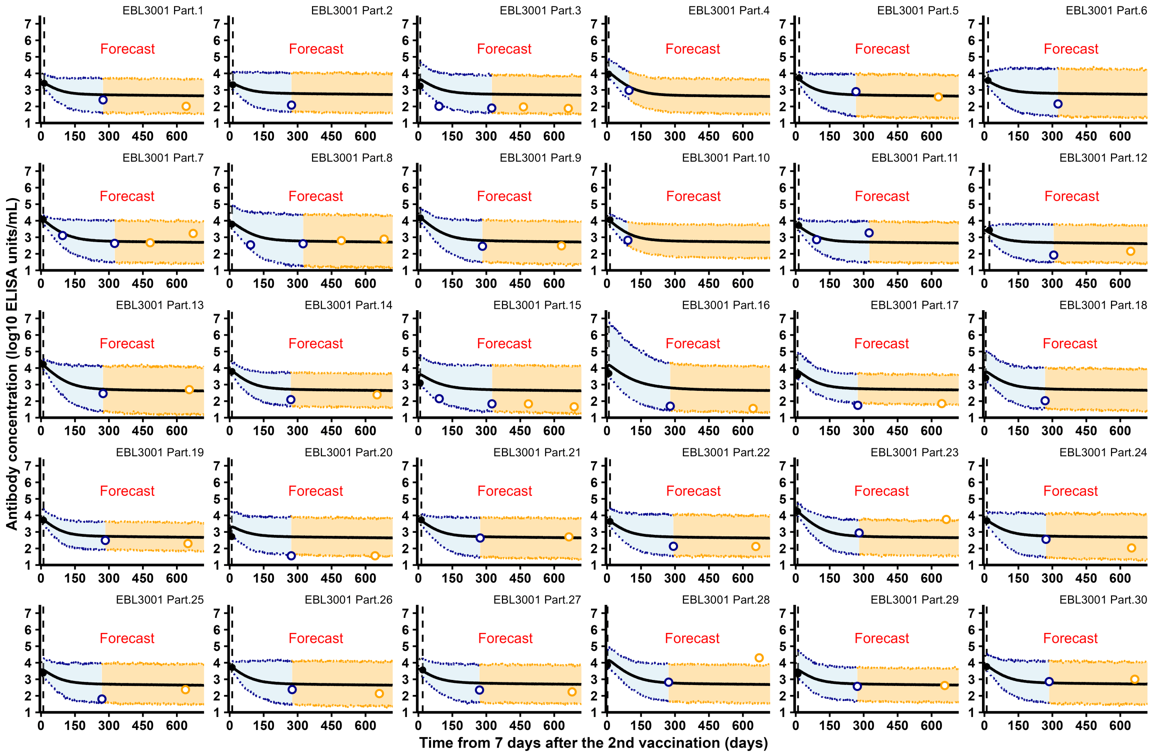

Supplementary Figure 3 – Individual antibody concentrations predicted by the model estimated on phase I data and EBEs evaluated using data restricted to the peak, for random sample of participants from EBL3001. Each sub-plot represents the individual antibody dynamics (in log10 ELISA units/mL) from 7 days after the 2nd vaccination. For each participant, the vertical dashed line represents the time limit (individual peak of dynamics) between the predictions (on the left) and the forecasts (short-term in blue and long-term in orange). Plain dots correspond to observations used to evaluate individual parameters while circles are observations not used in parameter estimation. Shaded areas correspond to 95% individual prediction intervals (accounting for the uncertainty on the individual parameter estimation and the measurement error) and the solid lines correspond to the prediction of the model.

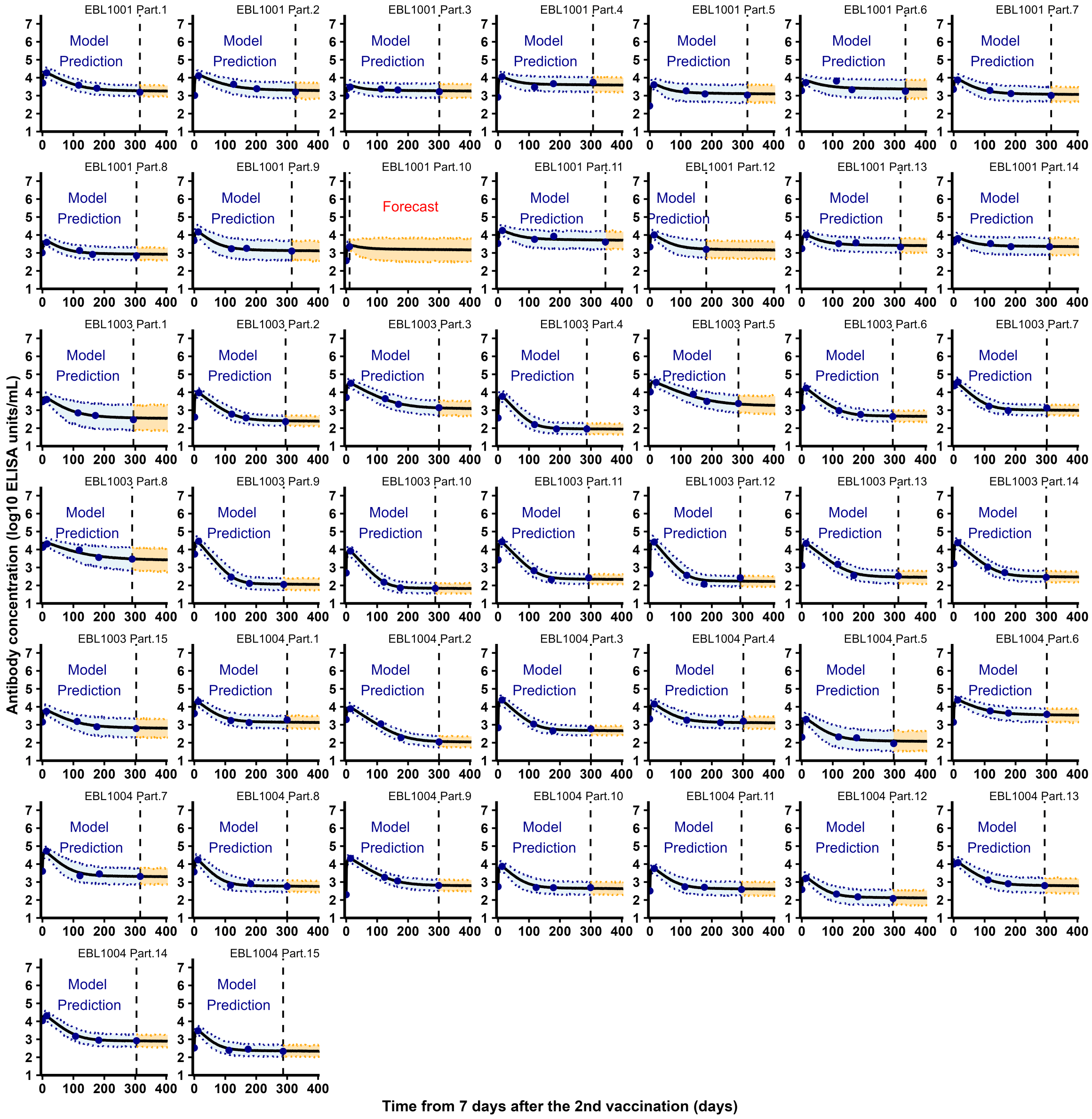

Supplementary Figure 4 – Individual antibody concentrations predicted by the model estimated on phase I data and EBEs evaluated using data restricted to 1 year, for Phase I participants. Each subplot represents the individual antibody dynamics (in log10 ELISA units/mL) from 7 days after the 2nd vaccination. For each participant, the vertical dashed line represents the time limit (1 year) between the predictions (on the left in blue) and the forecasts (on the right in orange). Plain dots correspond to observations used to evaluate individual parameters while circles are observations not used in parameter estimation. Shaded areas correspond to 95% individual prediction intervals (accounting for the uncertainty on the individual parameter estimation and the measurement error) and the solid lines correspond to the prediction of the model.

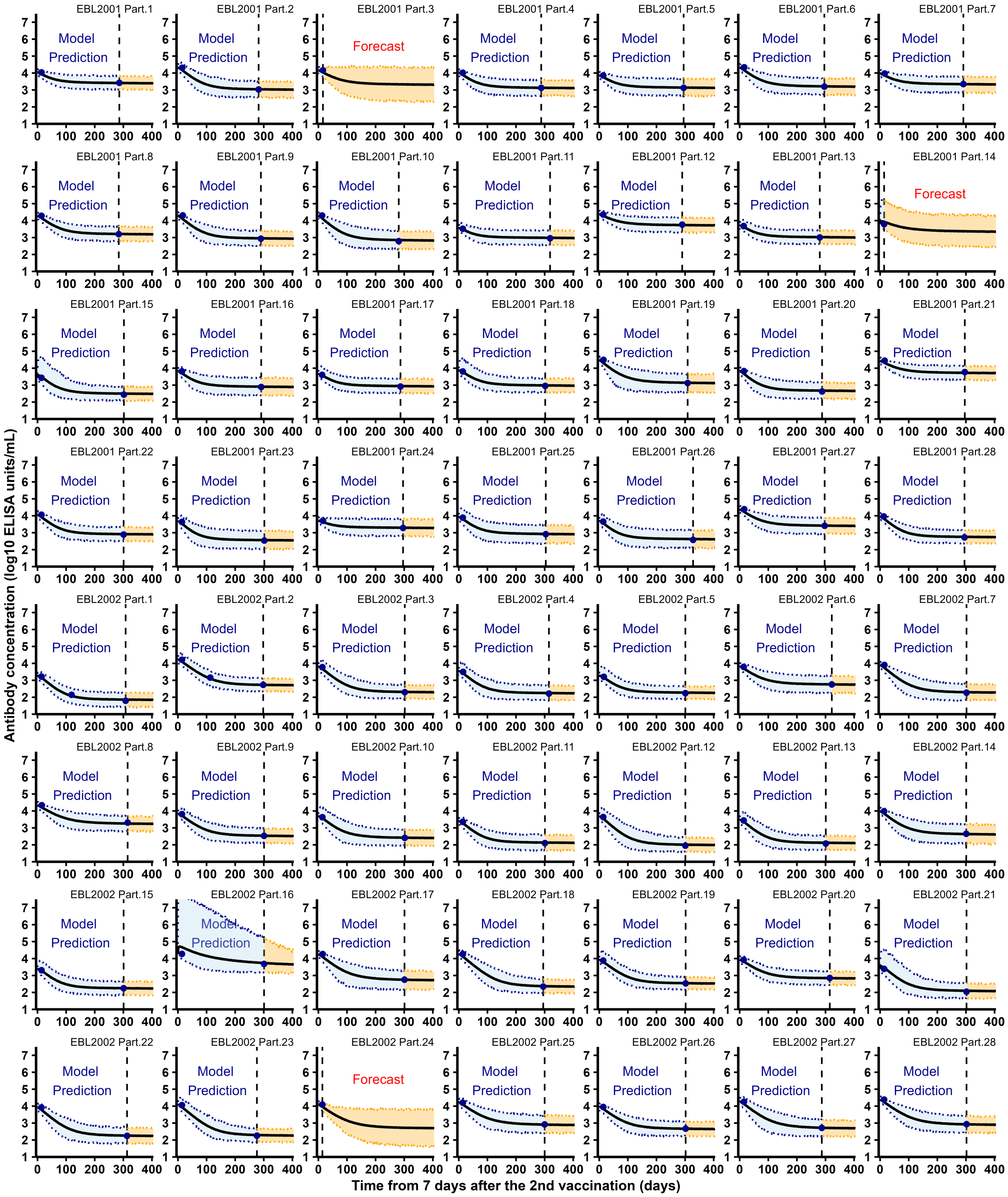

Supplementary Figure 5 – Individual antibody concentrations predicted by the model estimated on phase I data and EBEs evaluated using data restricted to 1 year, for random sample of participants from EBL2001 and EBL2002. Each subplot represents the individual antibody dynamics (in log10 ELISA units/mL) from 7 days after the 2nd vaccination. For each participant, the vertical dashed line represents the time limit (1 year) between the predictions (on the left in blue) and the forecasts (on the right in orange). Plain dots correspond to observations used to evaluate individual parameters while circles are observations not used in parameter estimation. Shaded areas correspond to 95% individual prediction intervals (accounting for the uncertainty on the individual parameter estimation and the measurement error) and the solid lines correspond to the prediction of the model.

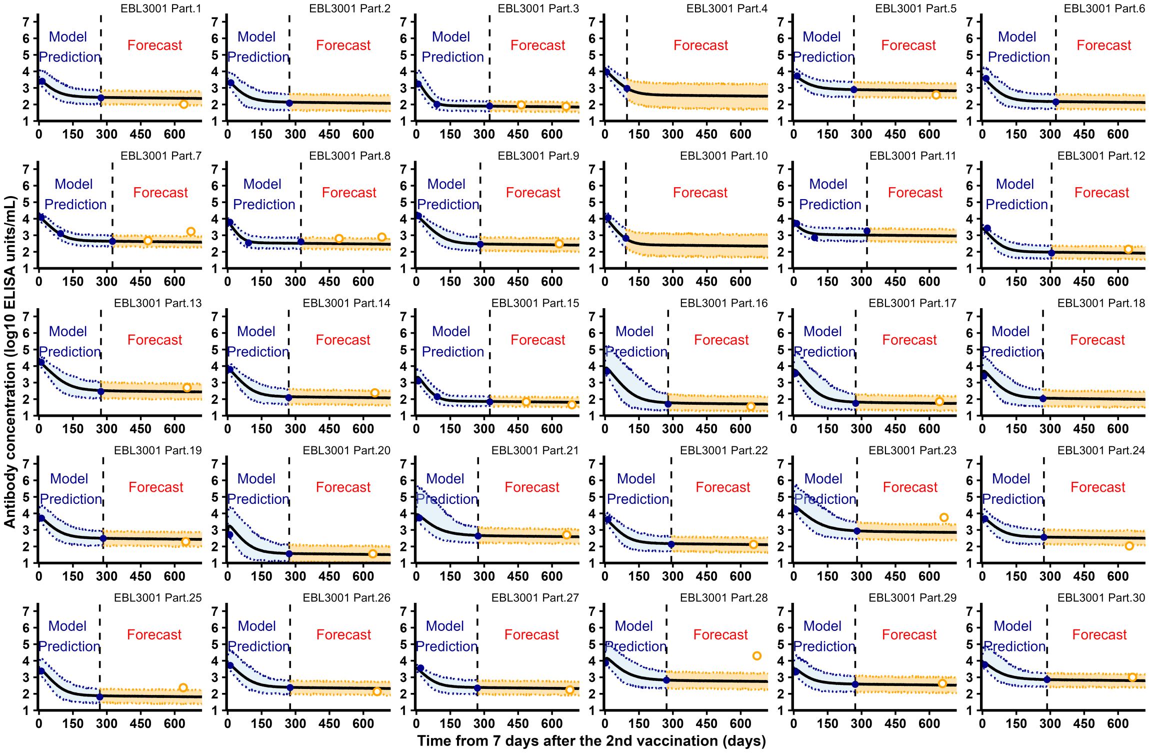

Supplementary Figure 6 – **Individual antibody concentrations predicted by the model estimated on phase I data and EBEs evaluated using data restricted to 1 year, for random sample of participants from EBL3001**. Each subplot represents the individual antibody dynamics (in log10 ELISA units/mL) from 7 days after the 2nd vaccination. For each participant, the vertical dashed line represents the time limit (1 year) between the predictions (on the left in blue) and the forecasts (on the right in orange). Plain dots correspond to observations used to evaluate individual parameters while circles are observations not used in parameter estimation. Shaded areas correspond to 95% individual prediction intervals (accounting for the uncertainty on the individual parameter estimation and the measurement error) and the solid lines correspond to the prediction of the model.

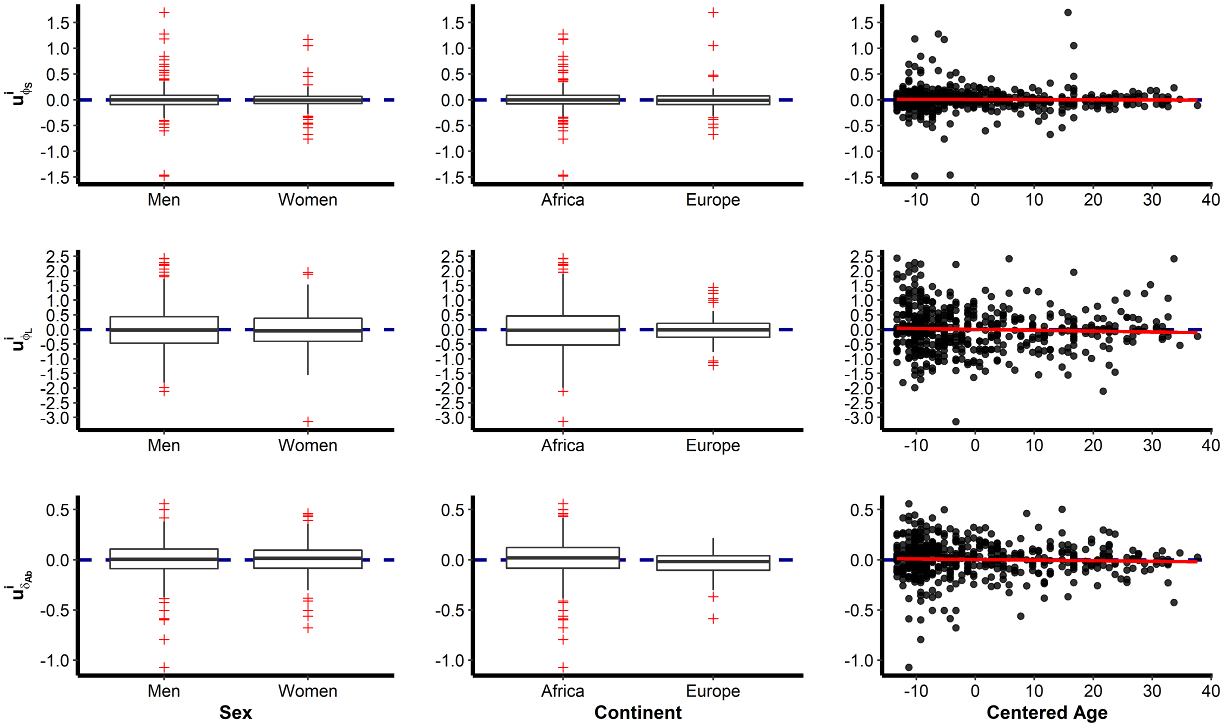

Supplementary Figure 7 – Random effects versus Covariates. Each subplot represents random effects (u^i) of a given parameter (1st row: ϕ_S, 2nd row: ϕ_L and 3rd row: δ_{Ab}) as a function of one of the three covariates selected in the model (Sex on the left, Continent in the middle, Centered Age on the right). Boxplots and dotplots were considered for categorical and continuous covariates, respectively. The horizontal blue dashed lines represent the threshold of zero and the red solid lines (3rd columns) represent the regression line.

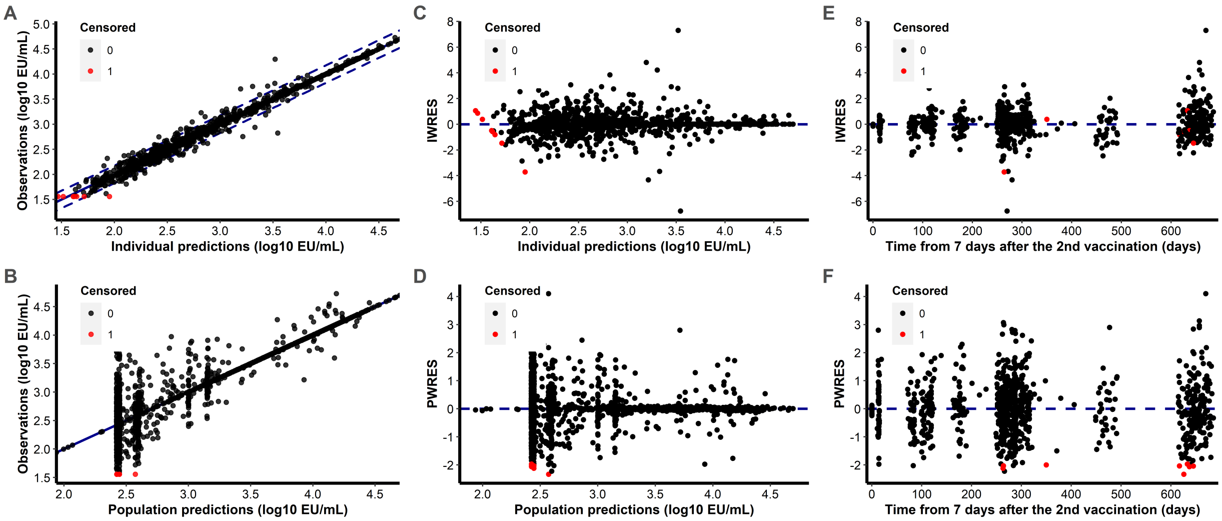

Supplementary Figure 8 – Plots of Goodness-of-fit of the mechanistic model estimated on Phase I and II data. (A) Observations versus individual predictions. A total of **95%** of observations falls within the **90%** confidence interval represented by the blue dashed slanted lines. (B) Observations versus population predictions. (C) Individual weighted residuals (IWRES) versus individual predictions. (D) Population weighted residuals (PWRES) versus population predictions. (E) IWRES versus time from 7 days after the second vaccination. (F) PWRES versus time from 7 days after the second vaccination. One each subplot, red and black dots represent censored and uncensored observations, respectively. Horizontal blue dashed lines represent the threshold of zero residuals.

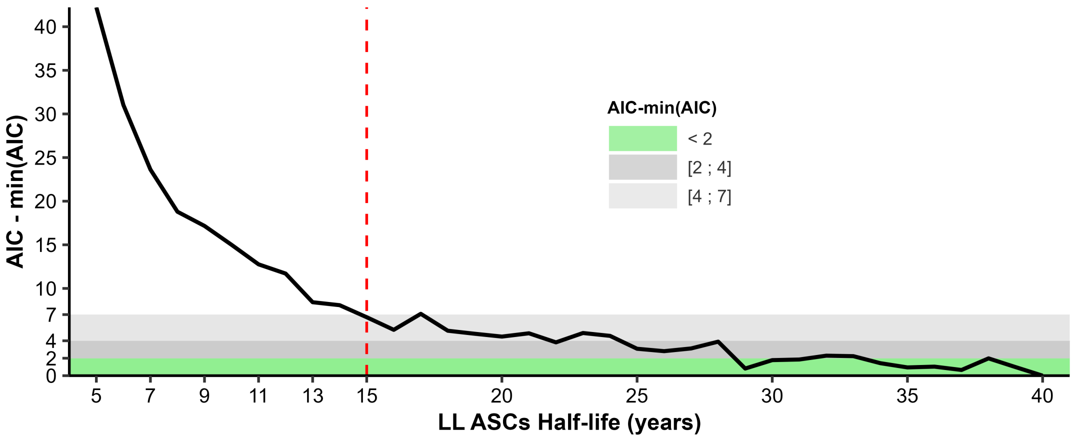

Supplementary Figure 9 – **Evaluation of ΔAIC$_m$ for all models with δ_L fixed at values corresponding to long-lived ASCs half-life ranging from 5 to 40 years.** Green, gray and light-gray areas highlight, respectively, intervals of ΔAIC$_m$ $\leqslant$ 2, 2 $\leqslant$ ΔAIC$_m$ $\leqslant$ 4 and 4 $\leqslant$ ΔAIC$_m$ $\leqslant$ 7. The vertical dashed line shows the first value of LL ASCs half-life for which ΔAIC$_m$ $\leqslant$ 7 (value used to fix the parameter δ_L in the article).

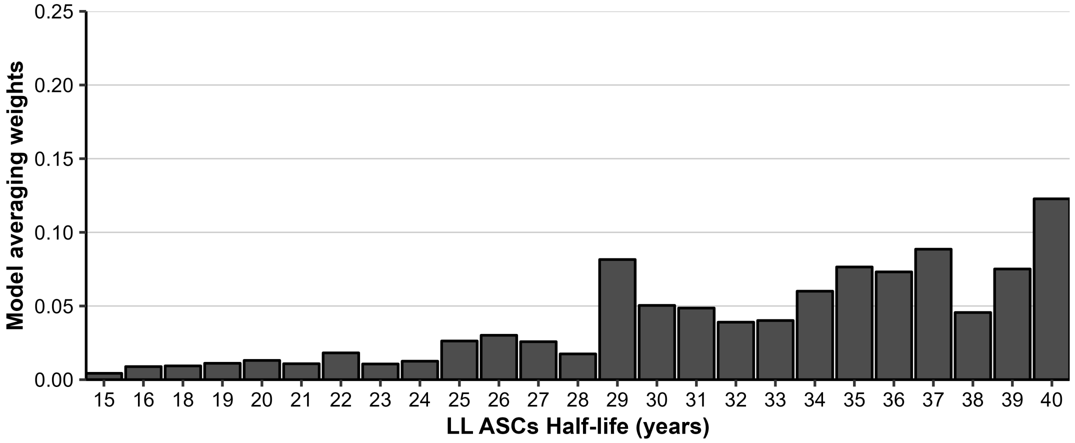

Supplementary Figure 10 – **Distribution of weights over the 25 candidates models involved in the model averaging**

Supplementary Tables

Supplementary Table 1 – Evaluation of the robustness and the quality of prediction of the model developed by Pasin et al. (5). The model was estimated on Phase I data and individual parameters were assessed, for each participant of Phase I and Phase II trials, using observation from 7 days post-second vaccination (day 64) to 1 year after the first vaccination.

	All trials	Phase I trials			Phase II trials		
		EBL1001	EBL1003	EBL1004	EBL2001	EBL2002	EBL3001
**Predictions from 7 days post-2nd vaccination to 1 year**							
RMSE[1]	0.045	0.077	0.062	0.058	0.020	0.027	0.046
Coverage (%)	100	100	100	100	100	100	100
Bias[1]	0.011	0.009	0.005	0.006	0.008	0.001	0.014
95% PI width	0.649	0.695	0.671	0.640	0.601	0.646	0.874
**Long-term forecast beyond 1 year**							
RMSE[1]	0.298						0.298
Coverage (%)	90.1						90.1
Bias[1]	-0.017						-0.017
95% PI width	0.874						0.874

CI: Confidence interval, PI: Prediction interval, RMSE: Root mean squared error. [1]Criteria calculated on the median of individual predictions.

Supplementary Table 2 – Parameter estimates obtained for the 25 candidate models involved in the model averaging, and the resulting model averaging estimators. Only estimates related to parameters δ_{Ab}, δ_S, Φ_S and Φ_L are provided.

$\log(2)/\delta_L$	δ_{Ab}		δ_S	ϕ_S		ϕ_L	
	Women	Men		Mean Age	Fold change	Africa	Europe
**15**	0.026 [0.023; 0.029]	0.036 [0.030; 0.043]	0.287 [0.232; 0.354]	3087 [2258; 4219]	0.939 [0.905; 0.974]	10.3 [9.15; 11.7]	39.4 [29.3; 53.0]
**16**	0.026 [0.023; 0.029]	0.035 [0.029; 0.042]	0.344 [0.334; 0.355]	3459 [2702; 4427]	0.930 [0.905; 0.956]	10.1 [8.97; 11.3]	39.4 [29.4; 52.8]
**18**	0.026 [0.023; 0.029]	0.036 [0.030; 0.043]	0.371 [0.336; 0.410]	3421 [2612; 4479]	0.934 [0.913; 0.956]	10.2 [9.03; 11.5]	37.9 [28.2; 51.0]
**19**	0.024 [0.021; 0.027]	0.034 [0.029; 0.041]	0.396 [0.388; 0.405]	3675 [2960; 4563]	0.939 [0.921; 0.957]	9.69 [8.62; 10.9]	35.0 [26.1; 46.9]
**20**	0.026 [0.023; 0.029]	0.036 [0.030; 0.044]	0.294 [0.278; 0.309]	2816 [2210; 3587]	0.939 [0.920; 0.958]	10.3 [9.07; 11.6]	38.5 [28.6; 51.7]
**21**	0.024 [0.022; 0.028]	0.034 [0.029; 0.041]	0.418 [0.413; 0.424]	3924 [3111; 4949]	0.941 [0.922; 0.960]	9.66 [8.59; 10.9]	35.3 [26.2; 47.6]
**22**	0.025 [0.022; 0.029]	0.036 [0.030; 0.043]	0.310 [0.303; 0.317]	3020 [2408; 3789]	0.942 [0.924; 0.961]	10.1 [8.96; 11.4]	36.6 [27.2; 49.3]
**23**	0.026 [0.023; 0.029]	0.035 [0.029; 0.042]	0.339 [0.333; 0.345]	3316 [2601; 4228]	0.951 [0.925; 0.979]	10.1 [8.97; 11.3]	37.2 [27.7; 49.9]
**24**	0.025 [0.022; 0.029]	0.036 [0.030; 0.044]	0.272 [0.240; 0.309]	2441 [1915; 3110]	0.947 [0.928; 0.965]	10.3 [9.10; 11.6]	37.2 [27.7; 49.8]
**25**	0.025 [0.022; 0.028]	0.035 [0.030; 0.042]	0.321 [0.313; 0.329]	3214 [2535; 4075]	0.944 [0.926; 0.963]	9.99 [8.89; 11.2]	36.5 [27.2; 49.1]
**26**	0.024 [0.021; 0.027]	0.034 [0.029; 0.041]	0.364 [0.351; 0.378]	3566 [2807; 4530]	0.944 [0.925; 0.965]	9.65 [8.57; 10.9]	35.2 [26.2; 47.3]
**27**	0.025 [0.022; 0.029]	0.036 [0.030; 0.043]	0.337 [0.318; 0.357]	3331 [2555; 4343]	0.946 [0.924; 0.968]	10.1 [8.95; 11.5]	37.0 [27.5; 49.7]
**28**	0.025 [0.022; 0.029]	0.036 [0.030; 0.043]	0.302 [0.288; 0.317]	2951 [2291; 3802]	0.949 [0.925; 0.974]	10.1 [8.93; 11.3]	36.1 [26.8; 48.5]
**29**	0.025 [0.022; 0.028]	0.035 [0.029; 0.042]	0.400 [0.394; 0.407]	4004 [3233; 4959]	0.939 [0.923; 0.956]	9.76 [8.67; 11.0]	35.5 [26.3; 47.9]
**30**	0.025[0.022; 0.028]	0.035 [0.029; 0.042]	0.346 [0.314; 0.381]	3367 [2654; 4271]	0.948 [0.929; 0.968]	9.72 [8.61; 11.0]	35.9 [26.7; 48.4]
**31**	0.024 [0.021; 0.027]	0.034 [0.029; 0.041]	0.342 [0.331; 0.353]	3268 [2578; 4142]	0.945 [0.925; 0.965]	9.57 [8.52; 10.8]	35.0 [26.1; 47.0]
**32**	0.025 [0.022; 0.029]	0.035 [0.029; 0.043]	0.289 [0.272; 0.306]	2734 [2077; 3599]	0.950 [0.922; 0.980]	9.99 [8.78; 11.4]	36.5 [27.0; 49.4]
**33**	0.025 [0.022; 0.028]	0.036 [0.030; 0.043]	0.280 [0.267; 0.293]	2748 [2192; 3444]	0.949 [0.928; 0.970]	9.97 [8.86; 11.2]	37.1 [27.7; 49.8]
**34**	0.025 [0.022; 0.029]	0.035 [0.029; 0.043]	0.286 [0.235; 0.348]	2590 [1946; 3446]	0.942 [0.920; 0.964]	9.97 [8.81; 11.3]	36.1 [26.8; 48.8]
**35**	0.025 [0.022; 0.028]	0.035 [0.029; 0.042]	0.370 [0.363; 0.378]	3572 [2807; 4545]	0.941 [0.922; 0.960]	9.75 [8.67; 11.0]	36.4 [27.1; 49.0]
**36**	0.025 [0.022; 0.028]	0.034 [0.028; 0.041]	0.342 [0.308; 0.380]	3255 [2566; 4130]	0.942 [0.924; 0.961]	9.51 [8.44; 10.71]	34.7 [25.8; 46.6]
**37**	0.025 [0.022; 0.028]	0.035 [0.029; 0.042]	0.385 [0.376; 0.394]	3472 [2776; 4344]	0.931 [0.914; 0.949]	9.74 [8.66; 11.0]	36.4 [27.1; 48.8]
**38**	0.025 [0.022; 0.028]	0.036 [0.030; 0.043]	0.394 [0.369; 0.421]	3750 [2882; 4480]	0.944 [0.923; 0.965]	9.88 [8.76; 11.1]	37.1 [27.6; 50.0]
**39**	0.025 [0.022; 0.028]	0.035 [0.030; 0.042]	0.294 [0.288; 0.301]	2943 [2377; 3644]	0.947 [0.930; 0.964]	9.82 [8.72; 11.1]	35.7 [26.6; 47.9]
**40**	0.026 [0.022; 0.029]	0.036 [0.029; 0.043]	0.314 [0.288; 0.341]	3175 [2427; 4153]	0.940 [0.914; 0.967]	9.86 [8.69; 11.2]	37.1 [27.5; 50.1]
**Model Averaging**	0.025 [0.022; 0.028]	0.035 [0.029; 0.042]	0.339 [0.272; 0.422]	3274 [2369; 4525]	0.942 [0.919; 0.966]	9.83 [8.67; 11.1]	36.2 [26.8; 48.9]

Supplementary Table 3 – Comparison of model parameters estimated on Phase I and II data for the model with a half-life of long-lived ASCs fixed by profile likelihood at 15 years (Model selection) or obtained by model averaging

		Model selection		Model Averaging	
**Parameter**	**Meaning**	**Mean**	**95% CI**	**Mean**	**95% CI**
**Fixed Effects**					
$\boldsymbol{\delta_{Ab}}$	antibody decay rate (day^{-1})				
Women		0.0251	[0.0223 ; 0.0283]	0.0250	[0.0220 ; 0.0284]
Men		0.0353	[0.0296 ; 0.0421]	0.0351	[0.0290 ; 0.0423]
$\mathbf{log}\,(2)/\boldsymbol{\delta_{Ab}}$	antibody half-life (days)				
Women		27.6	[24.5 ; 31.1]	27.8	[24.4 ; 31.6]
Men		19.6	[16.4 ; 23.4]	19.8	[16.4 ; 23.9]
$\boldsymbol{\delta_S}$	SL ASCs decay rate (day^{-1})	0.333	[0.326 ; 0.340]	0.339	[0.272 ; 0.422]
$\mathbf{log}\,(2)/\boldsymbol{\delta_S}$	SL ASCs half-life (days)	2.08	[2.04 ; 2.13]	2.05	[1.64 ; 2.55]
$\boldsymbol{\phi_S}$	SL ASCs influx (EU/mL/day)				
Mean Age (31.3 years)		3057	[2418 ; 3865]	3274	[2369 ; 4525]
FC ΔAge = + 1year[1]		0.934	[0.915 ; 0.954]	0.942	[0.919 ; 0.966]
$\boldsymbol{\phi_L}$	LL ASCs influx (EU/mL/day)				
African Part.		10.2	[9.01 ; 11.4]	9.82	[8.71 ; 11.1]
Eur. Part.		36.6	[27.3 ; 49.2]	36.2	[26.9 ; 48.7]
$\boldsymbol{\alpha}$	scaling factor - Lab effects				
α_{focus}		1.04	[0.93 ; 1.16]	1.06	[0.94 ; 1.19]
α_{Q2sol}		1.00	[0.98 ; 1.02]	1.00	[0.98 ; 1.02]
**Random Effects**					
$\boldsymbol{\omega_{\phi_S}}$	Sd of RE on ϕ_S	0.84	[0.56 ; 1.13]	0.80	[0.55 ; 1.05]
$\boldsymbol{\omega_{\phi_L}}$	Sd of RE on ϕ_L	0.88	[0.81 ; 0.96]	0.88	[0.81 ; 0.96]
$\boldsymbol{\omega_{\delta_{Ab}}}$	Sd of RE on δ_{Ab}	0.35	[0.29 ; 0.41]	0.36	[0.29 ; 0.42]
**Error Model**					
$\boldsymbol{\sigma_{Ab}}$	Sd of error model	0.107	[0.101 ; 0.112]	0.106	[0.101 ; 0.112]

CI: Confidence interval, EU: ELISA units, Eur.: European, FC: Fold change, LL ASCs: long-lived antibody secreting cells, Part.: Participants, RE: Random effects, SL ASCs: short-lived antibody secreting cells, Sd: Standard deviation. [1] Represents the multiplicative factor to apply to the value of ϕ_S, obtained for the mean age, for an increase in participant age of 1 year: ϕ_S(Mean Age + 1 year) = ϕ_S(Mean Age)× FC(ΔAge=+1). Therefore, the percentage of decrease of ϕ_S for a participant X years older than the mean age is given by 100×(1-FC(ΔAge=+1)^X).

Supplementary References

1. Burnham, K. P. & Anderson, D. R. Multimodel inference: understanding aic and bic in model selection. *Sociological methods & research* **33**, 261–304 (2004).
2. Gonçalves, A., Mentré, F., Lemenuel-Diot, A. & Guedj, J. Model averaging in viral dynamic models. *The AAPS Journal* **22**, 1–11 (2020).
3. Claeskens, G., Hjort, N. L. *et al.* Model selection and model averaging. *Cambridge Books* (2008).
4. Burnham, K. P., Anderson, D. R. & Huyvaert, K. P. Aic model selection and multimodel inference in behavioral ecology: some background, observations, and comparisons. *Behavioral ecology and sociobiology* **65**, 23–35 (2011).
5. Pasin, C. *et al.* Dynamics of the humoral immune response to a prime-boost ebola vaccine: quantification and sources of variation. *Journal of virology* **93**, e00579–19 (2019).
