# Supplementary material for: Prediction of long-term humoral response induced by the two-dose heterologous Ad26.ZEBOV, MVA-BN-Filo vaccine against Ebola
Source: NPJ Vaccines. 2023 Nov 8;8:174. doi: 10.1038/s41541-023-00767-y (PMC10632397; doi:10.1038/s41541-023-00767-y)
Supplement: Supplementary file 2 — Reporting Summary [file 41541_2023_767_MOESM2_ESM.pdf]

nature portfolio

Corresponding author(s): Thiébaut Rodolphe

Last updated by author(s): Aug 30, 2023

Reporting Summary

Nature Portfolio wishes to improve the reproducibility of the work that we publish. This form provides structure for consistency and transparency in reporting. For further information on Nature Portfolio policies, see our Editorial Policies and the Editorial Policy Checklist.

Statistics

For all statistical analyses, confirm that the following items are present in the figure legend, table legend, main text, or Methods section.

n/a	Confirmed	
☐	☒	The exact sample size (n) for each experimental group/condition, given as a discrete number and unit of measurement
☐	☒	A statement on whether measurements were taken from distinct samples or whether the same sample was measured repeatedly
☐	☒	The statistical test(s) used AND whether they are one- or two-sided<br>*Only common tests should be described solely by name; describe more complex techniques in the Methods section.*
☐	☒	A description of all covariates tested
☐	☒	A description of any assumptions or corrections, such as tests of normality and adjustment for multiple comparisons
☐	☒	A full description of the statistical parameters including central tendency (e.g. means) or other basic estimates (e.g. regression coefficient) AND variation (e.g. standard deviation) or associated estimates of uncertainty (e.g. confidence intervals)
☐	☒	For null hypothesis testing, the test statistic (e.g. F, t, r) with confidence intervals, effect sizes, degrees of freedom and P value noted<br>*Give P values as exact values whenever suitable.*
☒	☐	For Bayesian analysis, information on the choice of priors and Markov chain Monte Carlo settings
☒	☐	For hierarchical and complex designs, identification of the appropriate level for tests and full reporting of outcomes
☐	☒	Estimates of effect sizes (e.g. Cohen's d, Pearson's r), indicating how they were calculated

Our web collection on statistics for biologists contains articles on many of the points above.

Software and code

Policy information about availability of computer code

Data collection	*Provide a description of all commercial, open source and custom code used to collect the data in this study, specifying the version used OR state that no software was used.*
Data analysis	The original code (mlxtran models and R codes) developed in this work is available and free-of-cost on github (Inria SISTM Team) at the following link: https://github.com/sistm/ModelingEbola.git

For manuscripts utilizing custom algorithms or software that are central to the research but not yet described in published literature, software must be made available to editors and reviewers. We strongly encourage code deposition in a community repository (e.g. GitHub). See the Nature Portfolio guidelines for submitting code & software for further information.

Data

Policy information about availability of data

All manuscripts must include a data availability statement. This statement should provide the following information, where applicable:

- Accession codes, unique identifiers, or web links for publicly available datasets
- A description of any restrictions on data availability
- For clinical datasets or third party data, please ensure that the statement adheres to our policy

Janssen has an agreement with the Yale Open Data Access (YODA) Project to serve as the independent review panel for the evaluation of requests for clinical study reports and participant-level data from investigators and physicians for scientific research that will advance medical knowledge and public health. Data will be made

available following publication and approval by YODA of any formal requests with a defined analysis plan. For more information on this process or to make a request, please visit the Yoda Project site at http://yoda.yale.edu. The data sharing policy of Janssen Pharmaceutical Companies of Johnson & Johnson is available at https://www.janssen.com/clinical-trials/transparency.

Research involving human participants, their data, or biological material

Policy information about studies with human participants or human data. See also policy information about sex, gender (identity/presentation), and sexual orientation and race, ethnicity and racism.

Reporting on sex and gender	Findings on sex were found in the analysis.<br>Sex information was used as covariate in the modeling work
Reporting on race, ethnicity, or other socially relevant groupings	No such variables were used in our analysis
Population characteristics	Number of participants: 487<br>Sex - no. (%): 349 (72%) men ; 138 (28%) women<br>Age - mean (sd): 31.3 (12.4) years<br>BMI - mean (sd): 23.0 (3.9) kg/m2<br>Weight - mean (sd): 65.4 (11.7) kg
Recruitment	*Describe how participants were recruited. Outline any potential self-selection bias or other biases that may be present and how these are likely to impact results.*
Ethics oversight	[NCT02313077] The Phase I UK trial protocol and study documents were approved by the UK National Research Ethics Service.<br><br>[NCT02376426] The Phase I Kenya trial protocol and study documents were reviewed and approved by the local Ethics Committee and the Kenyan regulatory authority.<br><br>[NCT02376400] The Phase I Uganda/Tanzania trial protocol and study documents were reviewed and approved by the Tanzanian Medical Research Coordinating Committee of the National Institute for Medical Research, the Tanzania Food and Drugs Authority, the Uganda Virus Research Institute Research and Ethics Committee, the Uganda National Council for Science and Technology, the Uganda National Drug Regulatory Authority, and the Ethics Committee of the London School of Hygiene and TropicalMedicine.<br><br>[NCT02416453] The Phase II UK/France trial protocol and study documentswere approved by the French national Ethics Committee (CPP Ile de France III; 3287), the French Medicine Agency (150646A-61), the UK Medicines and Healthcare Products Regulatory Agency (MHRA), and the UK National Research Ethics Service (South Central,Oxford; A 15/SC/0211).<br><br>[NCT02564523] The Phase II Kenya/Uganda/Burkina Faso/Ivory Coast trial protocol and study documents were approved by local and national independent Ethics Committees and Institutional Review Boards.<br><br>[NCT02509494] The Phase II Sierra Leone trial protocol and study documents were approved by The study was approved by the Sierra Leone Ethics and Scientific Review Committee, the Pharmacy Board of Sierra Leone, and the London School of Hygiene & Tropical Medicine ethics committee.<br><br>The six trials were conducted in accordance with the principles of good clinical practice and the Declaration of Helsinki, and all participants gave formal, written consent before undergoing any trial-related procedure.

Note that full information on the approval of the study protocol must also be provided in the manuscript.

Field-specific reporting

Please select the one below that is the best fit for your research. If you are not sure, read the appropriate sections before making your selection.

☒ Life sciences ☐ Behavioural & social sciences ☐ Ecological, evolutionary & environmental sciences

For a reference copy of the document with all sections, see nature.com/documents/nr-reporting-summary-flat.pdf

Life sciences study design

All studies must disclose on these points even when the disclosure is negative.

Sample size	We considered data from six clinical trials : 3 Phase I and 3 Phase II trials<br>Phase I trials: a) EBL1001 [NCT02313077], b) EBL1003 [NCT02376426], c) EBL1004 [NCT02376400]<br>Phase II trials: a) EBL2001 [NCT02416453], b) EBL2002 [NCT02564523], c) EBL3001 [NCT02509494]<br><br>In Phase I trials, a total of 216 healthy adults were equally randomized into four vaccination regimens, with in each trial:<br>a) two with MVA-BN-Filo as first vaccination at day 1, followed by Ad26.ZEBOV on day 29 (n=18) or 57 (n=18)<br>b) two with Ad26.ZEBOV as prime vaccine on day 1, followed by MVA-BN-Filo on day 29 (n=18) or 57 (n=18)

Within each of these regimen, participants received either active vaccine or placebo in a 5:1 ratio.
In the phase I trial EBL1001, a fifth vaccination regiment was considered including 15 participants who received Ad26.ZEBOV as prime vaccine on day 1, followed by MVA-BN-Filo on day 15 (without placebo).

In the Phase II study EBL2001, 423 healthy adults participants were allocated into one of the three following vaccination regimen:
a) Ad26.ZEBOV as first vaccination at day 1, followed by MVA-BN-Filo on day 29
b) Ad26.ZEBOV as first vaccination at day 1, followed by MVA-BN-Filo on day 57
c) Ad26.ZEBOV as first vaccination at day 1, followed by MVA-BN-Filo on day 85
In addition, these participants were enrolled into 3 different cohorts:
1) Cohort 1: Participants received Ad26.ZEBOV and MVA-BN-Filo in an open-label fashion
2) Cohort 2: Participants were randomized to receive the 2-dose heterologous vaccine regimen or placebo in a 14:1 ratio
3) Cohort 3: Participants were randomized to receive the 2-dose heterologous vaccine regimen or placebo in a 10:3 ratio
In the Phase II study EBL2002, a total of 1075 participants were enrolled to receive one of the three following vaccination regimen:
a) Ad26.ZEBOV as first vaccination at day 1, followed by MVA-BN-Filo on day 29 [includes healthy adults, elderly participants and HIV-infected adults]
b) Ad26.ZEBOV as first vaccination at day 1, followed by MVA-BN-Filo on day 57 [includes healthy adults, elderly participants and HIV-infected adults]
c) Ad26.ZEBOV as first vaccination at day 1, followed by MVA-BN-Filo on day 85 [includes only healthy adults, elderly participants]
Participants were randomized to receive the 2-dose heterologous vaccine regimen or placebo in a 5:1 ratio.

In the study EBL3001, a total of 1023 participants (adults and children) were enrolled to receive Ad26.ZEBOV as first vaccination at day 1, followed by MVA-BN-Filo on day 57. Participants were randomized to receive the 2-dose heterologous vaccine regimen or placebo either in a 1:0 ratio (Stage 1), or in a 3:1 ratio (Stage 2).
In Stage 1, participants received a booster vacccination using Ad26.ZEBOV at 2 years post dose 1.

In our analysis, only participants from these six trials allocated to the vaccine regimen with Ad26.ZEBOV as first vaccination at day 1, followed by MVA-BN-Filo on day 57 were included (i.e, placebo were excluded). Moreover, in the study EBL2001, only participants from Cohorts 2 and 3 were included. Finally, only healthy adults were considered, excluding HIV-infected participants and children.
A total of 487 participants over the 6 studies were then included: 44 in Phase I trials, 71 in EBL2001, 137 in EBL2002 and 235 in EBL3001.

Data exclusions

We included only participants from the six trials receiving Ad26.ZEBOV as first vaccination on day 1 and MVA-BN-Filo as second vaccination on day 57 (Ad26/MVA D57). All participants receiving another vaccine regimen were excluded from the analysis. In particular, participants receiving placebo were excluded (n=168).

Moreover, only healthy adults were included in our analysis. Consequently, HIV-infected adults and children were excluded.

Finally, participants receiving their second vaccination outside the protocol-defined window were excluded: 57 +/- 1 day for Phase I trials and EBL2001, 57 +/- 3 days for EBL2002 and 57 +/- 7 days for EBL3001. Among the 725 healthy adults enrolled to receive Ad26/MVA D57, a total of 238 participants were excluded for not receiving their second dose (n=108) or outside the protocol-defined window (n=130).

Replication

Describe the measures taken to verify the reproducibility of the experimental findings. If all attempts at replication were successful, confirm this OR if there are any findings that were not replicated or cannot be reproduced, note this and describe why.

Randomization

All participants included in the analysis (n=487) were healthy adults receiving the same vaccine regimen: Ad26.ZEBOV as first vaccination on day 1, and MVA-BN-Filo as second vaccination on day 56. Among them, n=44 were enrolled in the 3 phase I trials (14 in EBL1001, 15 in EBL1003 and 15 in EBL1004), n=71 in the EBL2001 phase II trial, n=137 in the EBL2002 phase II trial and n=235 in the EBL3001 phase II trial.

Blinding

The six studies were conducted at least as observer-blinded studies.
EBL1001: Triple masking (Participant, Investigator, Outcomes Assessor)
EBL1003: Double masking (Participant, Investigator)
EBL1004: Double masking (Participant, Investigator)
EBL2001: Triple masking (Participant, Investigator, Outcomes Assessor)
EBL2002: Triple masking (Participant, Investigator, Outcomes Assessor)
EBL3001: Double masking (Participant, Investigator)

Reporting for specific materials, systems and methods

We require information from authors about some types of materials, experimental systems and methods used in many studies. Here, indicate whether each material, system or method listed is relevant to your study. If you are not sure if a list item applies to your research, read the appropriate section before selecting a response.

Materials & experimental systems

n/a	Involved in the study
☒	☐ Antibodies
☒	☐ Eukaryotic cell lines
☒	☐ Palaeontology and archaeology
☒	☐ Animals and other organisms
☐	☒ Clinical data
☒	☐ Dual use research of concern
☒	☐ Plants

Methods

n/a	Involved in the study
☒	☐ ChIP-seq
☒	☐ Flow cytometry
☒	☐ MRI-based neuroimaging

Clinical data

Policy information about clinical studies

All manuscripts should comply with the ICMJE guidelines for publication of clinical research and a completed CONSORT checklist must be included with all submissions.

Clinical trial registration

NCT02313077 ; NCT02376426 ; NCT02376400 ; NCT02416453 ; NCT02564523 ; NCT02509494

Study protocol

This paper being a meta-analysis, no study protocols were provided.

Data collection

Describe the settings and locales of data collection, noting the time periods of recruitment and data collection.

Outcomes

EBL1001 [NCT02313077], EBL1003 [NCT02376426] and EBL1004 [NCT02376400]: The purpose of these studies were to test the safety and immunogenicity of MVA-BN-Filo and Ad26.ZEBOV administered as heterologous prime-boost vaccine regimens in healthy adult participants.
Primary outcomes were then:
1) Number of participants with adverse events, up to 21 days post-boost
2) Number of participants with serious adverse events, up to the end of long-term follow-up
3) Number of participants with reactogenicity 1 week after each study vaccine administration

Secondary outcomes:
1) Immune responses to the study vaccine regimens as measured by a virus neutralization assay
2) Immune responses to the study vaccine regimens measured by an enzyme-linked immunosorbent assay (ELISA)
3) Immune responses to the study vaccine regimens as measured by an Enzyme-linked Immunospot Assay (ELISpot)

EBL2001 [NCT02416453]: The purpose of this study is to evaluate the safety, tolerability, and immunogenicity of 3 vaccination schedules of Ad26.ZEBOV and MVA-BN-Filo administered intramuscularly (IM) as 2-dose heterologous regimens.
Primary outcomes were then:
1) Number of participants with unsolicited adverse events, up to 42-day post dose 2
2) Number of participants with serious adverse events, up to the end of long-term follow-up.
3) Number of participants with immediate reportable events, up to the end of long-term follow-up.
4) Number of participants with solicited local adverse events at 7 days post-dose 1
5) Number of participants with solicited local adverse events at 7 days post-dose 2
6) Number of participants with solicited systemic adverse events at 7 days post-dose 1
7) Number of participants with solicited systemic adverse events at 7 days post-dose 2

Secondary outcomes:
1) Geometric mean concentrations (GMCs) of binding antibody levels against Ebola virus glycoprotein (EBOV GP) measured using Filovirus animal non-clinical group (FANG) enzyme-linked immunosorbent assay (ELISA) at 21-days post dose 2

EBL2002 [NCT02564523]: The purpose of this study is to assess the safety, tolerability and immunogenicity of three heterologous prime-boost regimens for Ebola vaccines Ad26.ZEBOV and MVA-BN-Filo. The study will include healthy adults and elderly participants, HIV infected participants and healthy children in 2 age strata.
Primary outcomes were then:
1) Number of participants with adverse events, up to 28 days post-dose 1
2) Number of participants with adverse events, up to 28 days post-dose 2
3) Number of participants with adverse events, up to 28 days post-dose 3
4) Number of participants with serious adverse events, up to the end of long-term follow-up
5) Number of participants with immediate reportable events, up to the end of long-term follow-up.
6) Number of participants with solicited local adverse events at 7 days post-dose 1
7) Number of participants with solicited local adverse events up to 7 days post-dose 2
8) Number of participants with solicited local adverse events up to 7 days post-dose 3
9) Number of participants with solicited systemic adverse events at 7 days post-dose 1
10) Number of participants with solicited systemic adverse events up to 7 days post-dose 2
11) Number of participants with solicited systemic adverse events up to 7 days post-dose 3

Secondary outcomes:
1) Geometric mean concentrations (GMCs) of binding antibody levels against Ebola virus glycoprotein (EBOV GP) measured using Filovirus animal non-clinical group (FANG) enzyme-linked immunosorbent assay (ELISA) at 21-days post dose 2

EBL3001 [NCT02509494]: The purpose of this study is the evaluation of the safety and immunogenicity of two candidate Ebola

vaccines Ad26.ZEBOV and MVA-BN-Filo, in a 2-dose heterologous regimen.
Primary outcomes:
1) Number of participants with solicited local adverse events at 7 days post-dose 1
2) Number of participants with solicited local adverse events at 7 days post-dose 2
3) Number of participants with solicited local adverse events at 7 days post-dose 3
4) Number of participants with solicited systemic adverse events at 7 days post-dose 1
5) Number of participants with solicited systemic adverse events at 7 days post-dose 2
6) Number of participants with solicited systemic adverse events at 7 days post-dose 3
7) Number of participants with serious adverse events, up to the end of long-term follow-up
8) Number of participants with unsolicited adverse events at 28 days post-dose 2
9) Number of participants with unsolicited adverse events, up to 42-day post dose 1
10) Number of participants with deaths, up to the end of long-term follow-up
11) Number of participants with immediate reportable events, up to the end of long-term follow-up.
Secondary outcomes:
1) Geometric mean concentrations (GMCs) of binding antibody levels against Ebola virus glycoprotein (EBOV GP) measured using Filovirus animal non-clinical group (FANG) enzyme-linked immunosorbent assay (ELISA) at 21-days post dose 2
